# Proteomic profiling reveals age-related changes in transporter proteins in the human blood–brain barrier

**DOI:** 10.1038/s41598-025-31224-6

**Published:** 2025-12-26

**Authors:** Xujia Zhou, Mina Azimi, Niklas Handin, Andrew Riselli, Bianca Vora, Eden Chun, Zilong Dang, Eric Huang, Sook Wah Yee, Per Artursson, Kathleen M. Giacomini

**Affiliations:** 1 Department of Bioengineering and Therapeutic Sciences, University of California, San Francisco, CA USA; 2 Department of Pathology, University of California, San Francisco, CA USA; 3 Department of Pharmacy, Uppsala University, Uppsala, Sweden

**Keywords:** Biochemistry, Cell biology, Neuroscience, Physiology

## Abstract

The Blood–Brain Barrier (BBB) is a selective barrier that regulates the entry of molecules including nutrients, environmental toxins, and therapeutic medications into the brain. Its function continues to evolve postnatally, through aging, and disease states. Here we present a global proteomics analysis focused on the ontogeny and aging of proteins in human brain microvessels (BMVs), predominantly composed of brain endothelial cells. Our proteomic study quantified 6,223 proteins and revealed possible age-related alterations in BBB permeability due to basement membrane component changes through the early developmental stage and age-dependent changes in transporter expression. Age dependent expression changes were observed within nutrient transporters and transporters that play critical roles in drug disposition. This research 1) provides important information on the mechanisms that drive changes in the metabolic content of the brain with age and 2) enables the creation of physiologically based pharmacokinetic models for CNS drug distribution across different life stages.

**Supplementary Information:**

The online version contains supplementary material available at 10.1038/s41598-025-31224-6.

## Introduction

The Blood–Brain Barrier (BBB) serves as a protective barrier that selectively regulates the entry of various substances into the Central Nervous System (CNS). Comprised mainly of brain endothelial cells, pericytes, astrocytic end-feet, and a basement membrane, the BBB protects the brain from pathogens, chemical carcinogen while allowing essential nutrients and therapeutic medications to enter^1^. The tight junctions between endothelial cells are one of the key components for proper BBB function, helping to significantly restrict the passive paracellular movement of small and large molecules^2^.

To maintain brain homeostasis, the BBB possesses a variety of transport systems, including active efflux transport, secondary active transport, and transcytosis (receptor-mediated and absorptive-mediated transport)^3^. Active efflux transporters, mostly represented by the ATP-binding cassette (ABC) family members ABCB1 (P-gp) and ABCG2 (BCRP), are highly expressed on the BBB and primarily prevent xenobiotic accumulation in the brain. As a result, many CNS-targeted drugs fail due to poor brain penetration caused by these transporters. Secondary active transporters mainly from the solute carrier (SLC) family, such as SLC2A1 (GLUT1) and SLC7A5 (LAT1), facilitate the uptake of crucial nutrients like glucose and amino acids. Deficiencies in these transporters are linked to developmental disorders, including GLUT1 deficiency syndrome and Brown-Vialetto-Van Laere syndrome^4^.

Despite the important role of these transporters in maintaining BBB integrity and homeostasis, research exploring their variation through lifespan is limited. Although most research relies on small cohorts or focuses on mRNA rather than protein levels^5–7^, increased evidence suggests that the BBB’s transport mechanisms adapt after birth and evolve with aging^8^, yet most pharmacological studies have been conducted only in adult patients. In fact, 50 to 90% of drugs prescribed to children or elderly are based on safety and efficacy data from clinical studies which only include adults under 65 years old, leading to a higher incidence of adverse drug reactions and therapeutic failures in these vulnerable populations^9,10^. While previous proteomic studies have examined the transporter profile in the BBB, these studies often had small sample sizes and limited their focus on adult and elderly populations^11–13^.

This study aims to provide a comprehensive analysis of protein expression changes at the BBB across three critical age stages—development, adulthood, and old age—focusing specifically on transporters, as their expression levels can significantly impact the pharmacokinetics of drugs in the brain as well as the brain metabolome. Using quantitative global proteomics, we revealed key age-dependent shifts in protein expression and highlighted critical protein networks associated with BBB function throughout the lifespan. These findings address knowledge gaps in BBB protein dynamics during both development and aging, contributing to our understanding of how these changes impact the brain metabolome and inform drug development for central nervous system (CNS) disorders.

## Results

### Experimental workflow and overview of brain microvessel (BMV) proteome

**Fig. 1:**
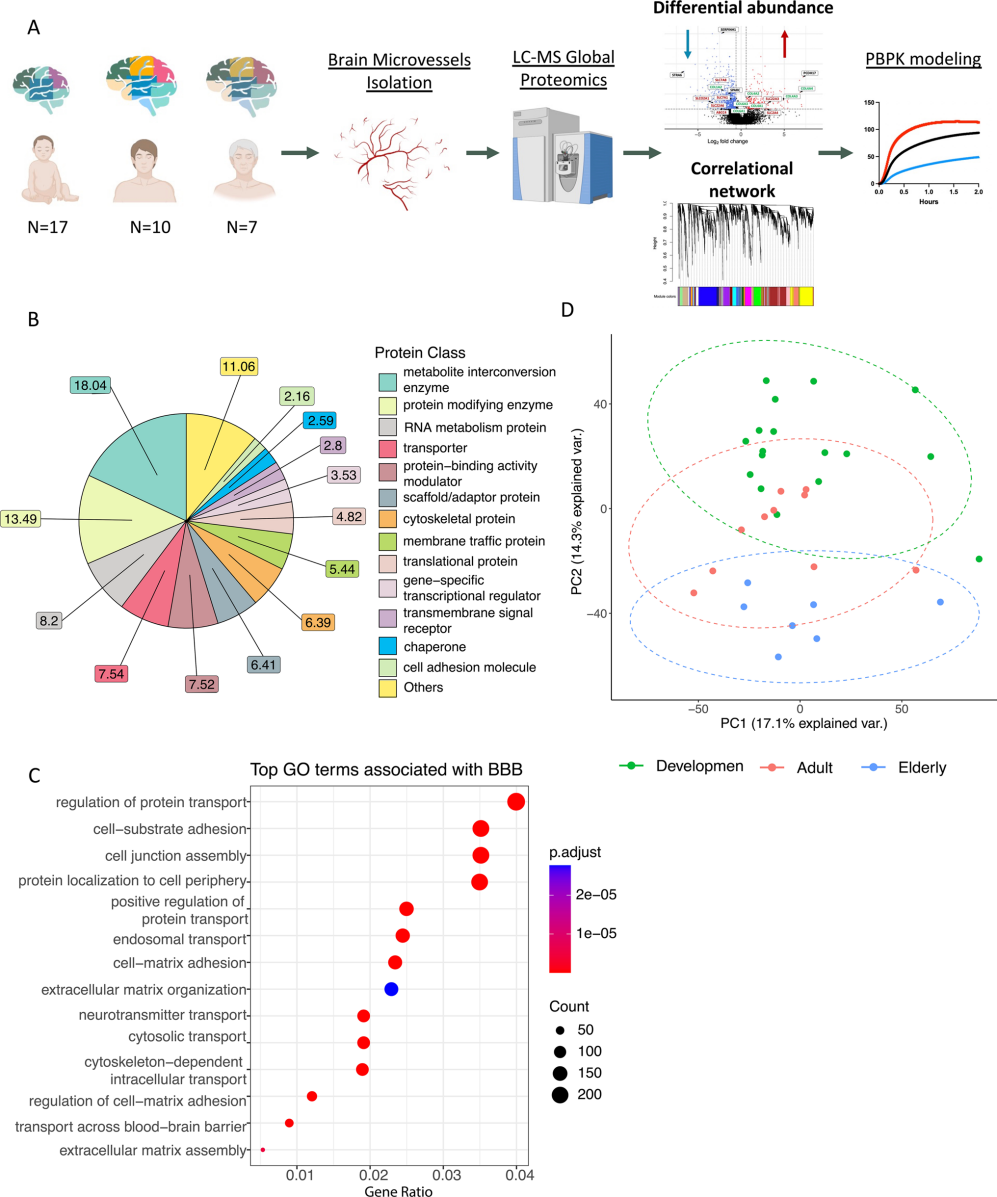
Brief experimental workflow and overview of BMV proteome. (**A**) Brief experimental workflow. Brain microvessels (BMVs) were isolated from frozen insular cortical brain tissue, which were then digested and analyzed using LC–MS/MS proteomic methods. Differential protein analysis and weighted correlation network analysis(WGCNA) were performed to identify proteins that differ between age groups and possible altered blood brain barrier parameters were incorporated into physiologically based pharmacokinetic (PBPK) modeling simulations. (**B**) The PANTHER protein class of the identified proteins in BMV proteome. (**C**) Top BBB related GO terms associated with our BMV proteome are shown. X-axis is the gene enrichment ratio (Gene ratio) and the bubble size indicates the numbers of proteins associated a biological process GO term, with color maps the FDR value (p.adjust, q-value) of the enrichment analysis. (**D**) Principal component analysis (PCA) on BMV proteomic dataset showing the separation between different age groups.

Global proteomic analysis using LC–MS/MS global proteomics was conducted on BMVs isolated from thirty-four healthy brain specimens, collected from neonates to elderly individuals, as well as from three samples from AD patients (Supplemental Table 1). For the analysis, we categorized the brain samples into three age groups: developmental, adult, and elderly (Supplemental Table 1). A brief overview of our study’s workflow is illustrated in Fig. 1A. In total, our BMV proteome includes 6,223 proteins and is distributed across 13 distinct protein classes, including transporters (7.54%) and cell adhesion molecules (2.16%) (Fig. 1B)^14^. GO enrichment analysis of this BMV proteome revealed BBB-associated GO terms such as *cell substrate adhesion*, *cell junction assembly*, and *transport across the BBB* (Fig. 1C). Major tight junction proteins and integrins (e.g. CLDN5, ITGB1, TJP1) are expressed in our BMV proteome. Claudin-11, which has been shown to colocalize with CLDN5, exhibits significantly lower expression in Development group (Supplemental Fig. 1)^15^. An unsupervised principal component analysis (PCA) was conducted to explore the underlying variations within our dataset. PCA and the sample clustering dendrogram of our BMV proteome showed no obvious discrepancy between the samples (Supplemental Fig. 2A). We found that the BMV proteome can be effectively categorized into the Development and Elderly groups based on the first two principal components (PCs), with the Adult group positioned in between (Fig. 1D). The PCA loading plots allowed us to identify proteins related to the observed clustering. These proteins, including cytoskeletal-associated protein 4 (CKAP4) and C-X-C motif chemokine ligand 12 (CXCL12), play key roles in regulating BBB permeability (Supplemental Fig. 2B)^16,17^ and showed significant age-related correlations in our BMV proteome (Supplemental Fig. 2C).

### BMV proteome reveals expression patterns that are indicative of brain development stages

**Fig. 2:**
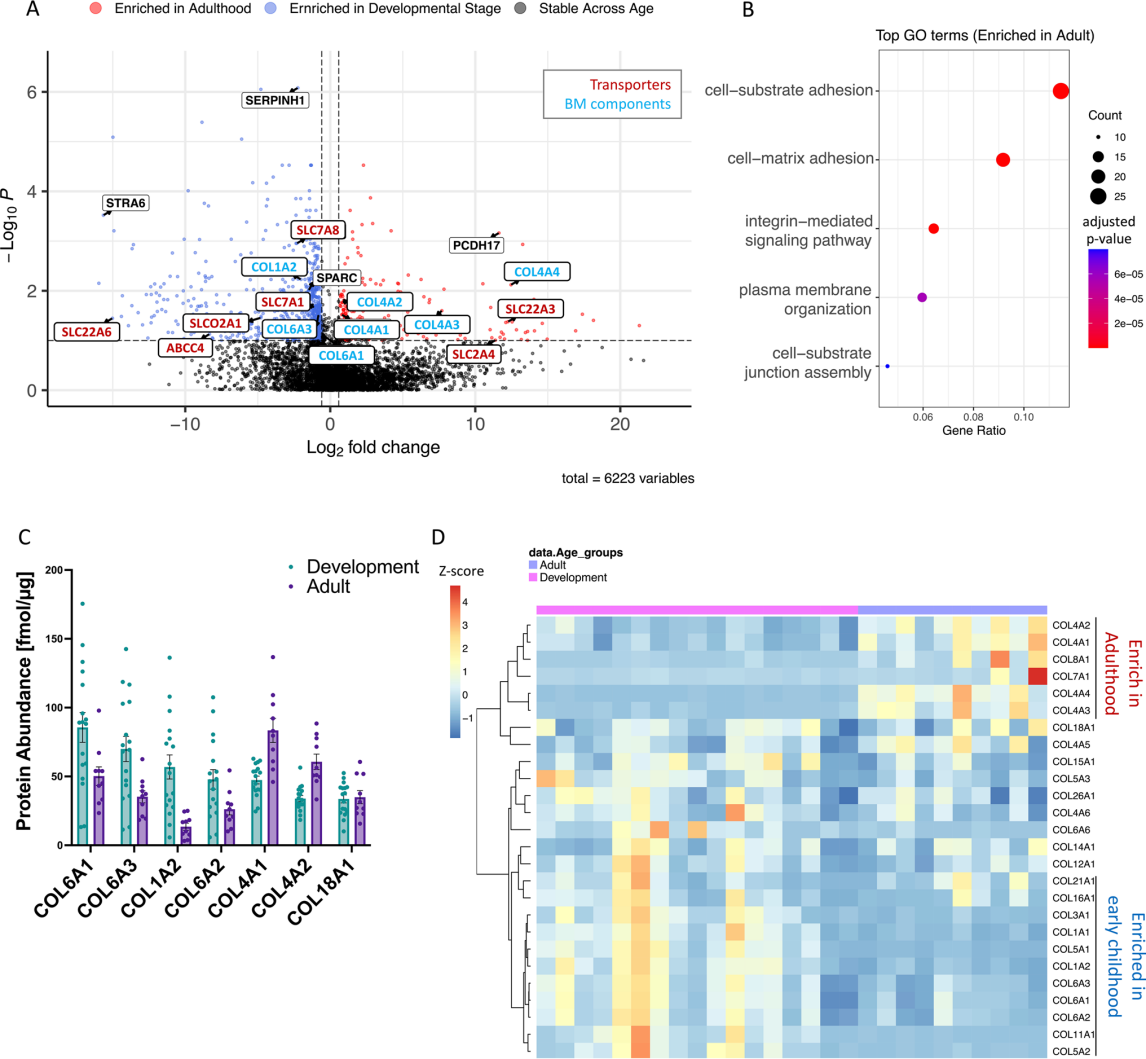
Differential expression of discovery BMV proteome through early childhood. (**A**) Volcano plot displaying the log2 fold change (x axis) against the t test-derived -loglO statistical *P* value (y axis) for all proteins differentially expressed between Development group (N = 17) and Adult group (N = 10) of the BMV proteome. Proteins with significantly decreased levels in Adults (*P* < 0.1) are shown on the left side, while the proteins with significantly increased levels through developement are shown on the right side. Transporters are labeled in red and proteins important for BBB integrity are labeled in green. (**B**) Top GO terms associated with proteins significantly increased with age are shown. X-axis is the gene enrichment ratio (Gene ratio) and the bubble size indicates the numbers of proteins associated with a biological process GO term, with color mapping the FDR value (adjusted *p *value) of the enrichment analysis. (**C**) Bar graph showing collagens highly expressed in the BMV proteomic dataset, ordered by expression levels with the highest in the Development group. (**D**) Protein expression of collagens which are expressed in our proteomic dataset and exhibit positive(red) or negative(blue) correlation with age. Scale represents the row Z-score (each row presents one protein), which is calculated by taking each individual’s protein expression, subtracting the mean expression, and then dividing by the standard deviation of that protein.

Differential expression was assessed using a statistical t-test analysis; more specifically, we aimed to identify proteins with significantly altered abundance levels (Absolute Log2 Fold Change > Log2(1.5), *P* < 0.1) between the Development and Adult groups. There was a total of 240 proteins with significantly increased abundance as individuals transition into adulthood (Fig. 2A, red circles). GO enrichment analysis of these proteins underscored their substantial link to BBB functionality, illustrating their involvement in key biological processes like *cell substrate adhesion*, *cell matrix adhesion*, and *the regulation of metal ion transport* (Fig. 2B). COL4A1 and COL4A2, which encode the alpha1 and alpha2 chains of type IV collagen respectively, are known to be the major components of the basement membrane and play crucial roles in the stabilization and maintenance of blood–brain barrier (BBB) integrity^1^. Interestingly, while these two proteins were also the most abundant collagens in adulthood within our dataset, we found a significantly increased expression with age, especially as individuals matured into adulthood (COL4A1, *P* value = 0.030; COL4A2, *P* value = 0.017) (Fig. 2A). Consequently, a notable shift was observed in the dominance of collagen families; specifically, Collagen VI (COL6A1, *P* value = 0.074; COL6A3, *P* value = 0.031) was identified as the predominant collagen during the developmental stages (Fig. 2C, Supplemental Fig. 3). Although the roles of COL4A3 (*P* value = 0.025) and COL4A4 (*P* value = 0.0076), whose dysfunction leads to renal disease, have not been extensively explored in the brain, both proteins are significantly enriched in the Adult group (19.6-fold and 117.1-fold increase respectively) . Our BMV proteome identified 29 collagens, with 37.9% (11 collagens) showing a negative correlation with age and 24.1% (7 collagens) exhibiting an enrichment in adulthood (Fig. 2D).

Beyond collagens, we also examined the expression of other key basement membrane proteins in our dataset (Supplemental Fig. 3). We identified nine laminins in our dataset, with significant age-related correlation and perlecan (HSPG2) was significantly upregulated during development. These findings indicate that alterations in basement membrane composition during development may influence BBB permeability, potentially affecting passive diffusion and selective barrier function as individuals age.

Conversely, there were a total of 757 proteins that were significantly enriched in developmental stage (Fig. 2A, blue circles). These included SERPINH1 (a collagen-specific ER chaperone; *P* value = 8.31 × 10^−7^) and SPARC (an extracellular chaperone required for spatial assembly of collagen IV; *P* value = 0.00964)^18^. This decrease further supports the dynamic development of the basement membrane during early childhood. Moreover, GO analysis revealed that the proteins that were decreased through development were predominantly involved in *extracellular matrix organization* and *mRNA processing* (Supplemental Fig. 4A). A similar trend has been observed prenatally^6^. Our results here support that BBB development is still ongoing after birth and that the metabolic process of RNA in early childhood is different from adults.

### Network analysis of BMV proteome reveals modules linked to barrier integrity and the BBB transport system

**Fig. 3:**
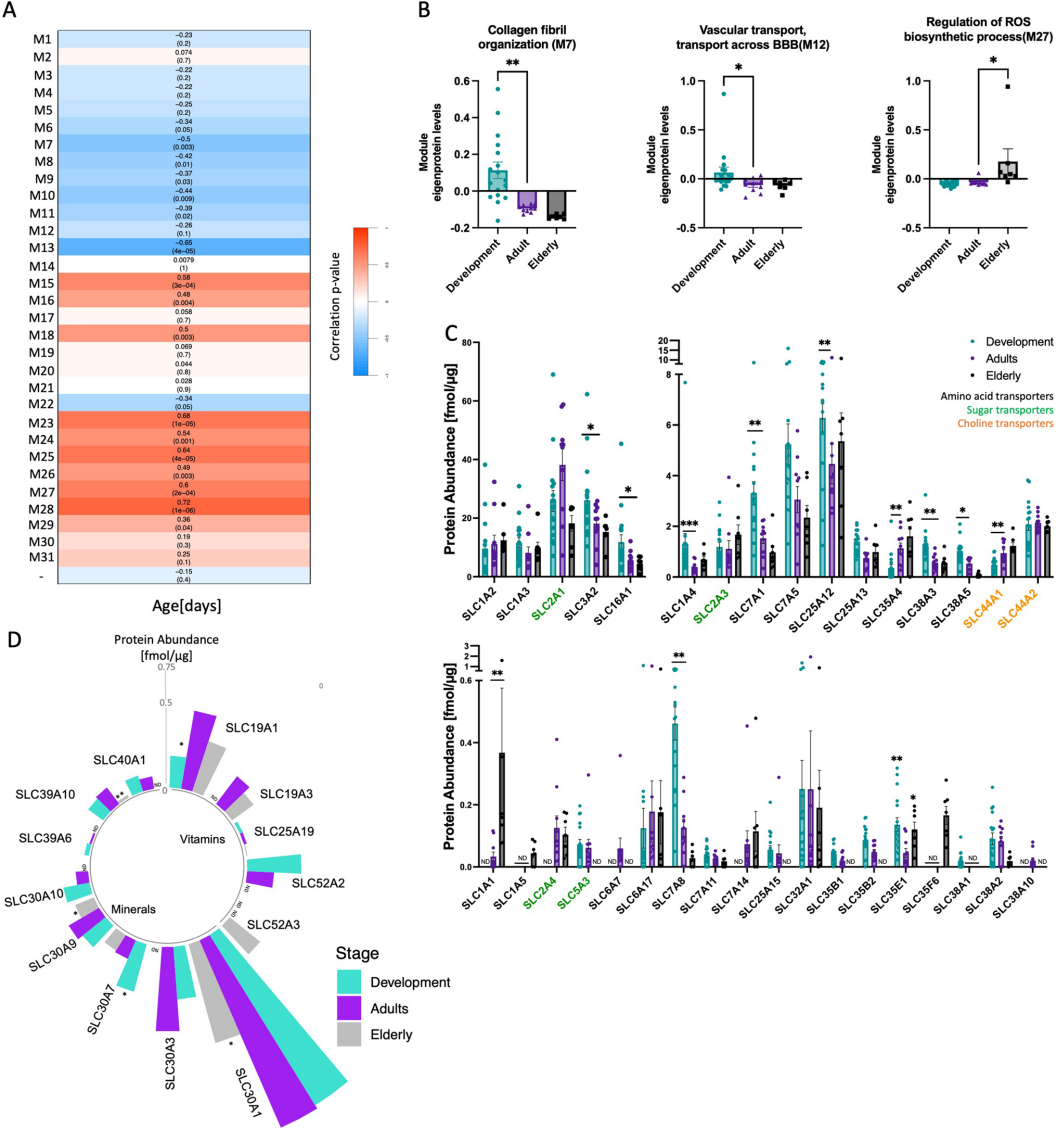
BMV proteins co-expression network identified age-dependent modules which are important for BBB function. (**A**) The correlation between modules and age. Heatmaps show the correlation between eigengene and age and each cell contains the corresponding correlation followed by *p-*value. (**B**) Module eigenprotein levels by age groups (Development, Adult, Elderly) for the three blood brain barrier related modules. Modules are grouped by different age groups. (**C**) Macronutrient transporters and transporters involving in macronutrient metabolism in our BMV proteomic dataset are shown in bar graphs. Amino acid transporters are labeled as black, sugar transporters are labeled as green and choline transporters are labeled as orange. (**D**) Micronutrient transporters in our BMV proteomic dataset are shown in a circular bar graph, bars represent the mean of the transporter expressions in different age groups. For the bar graphs, Kruskal–Wallis tests followed by Dunn’s post hoc test were used to compare the mean of each age groups with the mean of the Adult group. Data are represented as mean ± SEM and each point represents one sample. *P < 0.05, **P < 0.01, ***P < 0.001. ND, not detected in more than 30% of samples in specific age group.

We subsequently performed a network analysis on the discovery BMV proteome using the WGCNA algorithm, which organizes the dataset into modules of proteins with similar expression patterns^19^. WGCNA is often used to understand the relationships between gene co-expression networks, individual genes, and sample traits with high sensitivity. It is particularly effective at identifying proteins or genes with subtle fold changes or lower abundance, which might otherwise be overlooked in traditional analysis. The WGCNA identified 30 modules of co-expressed proteins and the biology represented by each module was determined using network analysis provided by Cytoscape^20^ (https://cytoscape.org/, Supplemental Table 6). To assess whether a module was related to the age shift, we correlated each module eigenprotein, or the first principal component (PC) of module protein expression, to each sample’s specific age (in days). We observed that 10 modules show a significant positive correlation (> 0.1) and 7 modules show a significant negative correlation (< − 0.1) through the early development to elderly stages (Fig. 3A). GO terms associated with each module are provided in Supplemental Data File 1. Since our BMV proteome includes samples from a large range of ages, and many proteins do not have a linear change throughout a person’s lifetime, in order to further dissect how these modules are altered through development and in the aging process respectively, we compared the module eigenprotein across age groups (Development, Adult, and Elderly). The module linked to collagen fibril organization (M7) which includes basement membrane components such us COL1A2 (Collagen type I alpha 2 chain), COL11A1 (Collagen type XI alpha 1 chain), and LUM (Lumican), shows significantly enriched expression in the Development group (Fig. 3B). This is consistent with the GO enrichment analysis we did utilizing the differentially expressed proteins among the Development and Adult groups. We also identified a significant alteration in the module linked to transporters across the BBB (M12) during early developmental stages, with nutrient transporters such as the amino acid transporters, SLC7A1 (CAT1), SLC7A5 (LAT1), and the docosahexaenoic acid transporter, SLC59A1 (MFSD2A), exhibiting age-dependent changes (Supplemental Table 2).

### Nutrient transporter expression in the BBB shows age dependence

The human brain goes through significant changes postnatally. While a newborn already has most of its lifelong neurons, the brain doubles in size in the first year due to an accelerated rate of myelination and the formation of synapses during early childhood^21^. Proper nutrition and availability of nutrients to the CNS are crucial for normal brain and neurocognitive development. We identified a total of 56 SLCs involved in nutrient transport in the human BMV proteome. Twenty-five of the 56 have been quantified for the first time through a global proteomic analysis of the human BMVs. Moreover, our proteomic analysis revealed that the BBB, which controls the delivery of essential nutrients to support brain and neuron development, has differential protein expression during early development, including that of multiple nutrient transporters. Amino acids such as l-arginine and l-serine are required for regulating synapse formation/patterning and neurotransmitter synthesis^22^. In our dataset, we found that l-arginine transporter SLC7A1 and glutamate/l-serine SLC38A5 (SNAT5), were highly expressed in early childhood, with expression levels decreasing in adulthood (Fig. 3C). Additionally, SLC3A2 (CD98hc/4F2hc) and SLC7A5, the two most highly expressed amino acid transporters in our BMV proteome, exhibit a significant negative correlation with age (Supplemental Fig. 5).

Micronutrients such as vitamins and minerals are crucial for normal neurodevelopment. The riboflavin transporter, SLC52A2 (Km = 0.33 µM) was only detected in Development and Adult groups, while another riboflavin transporter, SLC52A3 (Km = 0.98 µM), which is in the same family but has a slightly higher Michaelis–Menten constant (Km), was only expressed in Elderly group (Fig. 3D)^23,24^. These data suggest that riboflavin delivery to the brain may change depending on age, with the increased delivery occurring during development. Interestingly, while zinc transporters are among the most highly expressed micronutrient transporters in BMV throughout life, SLC30A1, the most abundant zinc transporter, shows significantly lower expression in the elderly population. Recently identified as the primary transporter for choline uptake into the brain, SLC49A2 has been found in BMVs in both Development and Adult groups but was undetected in the Elderly group (Supplemental Table 2)^25^. These observations suggest that delivery of specific macro- and micro-nutrients to the brain changes with maturation and aging.

Malfunctions in these nutrient transporters can result in severe neurological outcomes. Approximately 20% of known human SLC transporters are linked to Mendelian diseases, with 39 identified in our BMV proteome^26^. Many disease-associated transporters are prominently expressed during early childhood, highlighting their roles in neurodevelopment (Table 1). For instance, SLC1A4, a serine transporter implicated in spastic tetraplegia and microcephaly (OMIM# 616657), was significantly enriched in the Development group and dropped by 70% in adulthood (Fig. 3D, Table 1, Dunn’s multiple comparison test *P* value (Dunn’s *P* value) = 0.042). SLC16A2, a thyroid hormone transporter associated with Allan-Herndon-Dudley syndrome (OMIM# 300523), showed highest expression in early life (Dunn’s *P* value = 0.0041) and exhibited a sequential reduction with age. (Table 1).

**Table 1:** SLC transporters on BBB associated Mendelian Disease.

Gene (Protein)	Predominant substrate(s)	Mendelian disease(s)(#OMIM)	Protein abundance [pmol/mg]				
			Development	Adult	Elderly	Developmental Changes* (*P* value)	Aging Changes* (*P* value)
SLC1A1 (EAAT3)	L-Glu, D/L-Asp	Dicarboxylic aminoaciduria (222730), Schizophrenia susceptibility 18 (615232)	ND	0.033	0.367	↑	0.160
SLC1A2 (EAAT2)	L-Glu, D/L-Asp	Developmental and epileptic encephalopathy 41 (617105)	9.621	11.144	12.447	0.696	0.733
SLC1A3 (EAAT1)	L-Glu, D/L-Asp	Episodic ataxia, type 6 (612656)	11.564	8.108	9.718	0.213	0.590
SLC1A4 (ASCT1)	L-Ala, L-Ser	Spastictetraplegia, thincorpus callosum, and progressive microcephaly (SPATCCM, 616657)	1.305	0.393	0.692	↓(0.042)	0.065
SLC2A1 (GLUT1)	Glucose	GLUT1 Deficiency Syndrome 1 (606777)/ Syndrome 2(612126), Stomatin-deficient cryohydrocytosis with neurologic defects(608885), Dystonia 9 (601042)	25.855	38.187	18.229	0.075	↓(0.0059)
SLC4A1(AE1)	Chloride cicarbonate	Cryohydrocytosis(185020), Distal renal tubular acidosis 1(179800)	48.525	39.659	25.713	0.510	0.210
SLC4A4 (NBCe1)	Sodium bicarbonate	Renal tubular acidosis, proximal, with ocular abnormalities(604278)	0.448	0.408	0.431	0.809	0.887
SLC5A6 (SMVT)	Biotin, lipoate panthothenate	Peripheral motor neuropathy, childhood-onset, biotin-responsive(619903), Sodium-dependent multivitamin transporter deficiency(618973)	0.776	0.726	0.664	0.786	0.803
SLC6A1 (GAT1)	GABA	Myoclonic-atonic epilepsy (616421)	1.854	2.972	1.630	↑(0.0212)	↓(0.0264)
SLC6A17 (NTT4)	NAAs	Intellectual developmental disorder, autosomal recessive 48 (616269)	0.125	0.178	0.176	0.655	0.988
SLC7A14	CAAs	Retinitis pigmentosa 68 (615725)	ND	0.073	0.115	0.329	0.602
SLC9A1 (NHE1)	Na + , Li + , H + , NH4 +	Lichtenstein-Knorr syndrome(616291)	0.867	1.068	0.852	0.179	0.366
SLC9A6 (NHE6)	Na + , K + , H +	Intellectual developmental disorder, X-linked syndromic, Christianson type(300243)	0.064	ND	0.057	↓	↑
SLC12A5 (KCC2)	K + , Cl-	Developmental and epileptic encephalopathy 34(616645)	0.367	0.755	1.200	0.422	0.504
SLC12A6 (KCC3)	K + , Cl-	Agenesis of the corpus callosum with peripheral neuropathy (218000)	0.022	0.027	0.006	0.683	0.125
SLC13A5 (Nac2)	Citrate, succinate	Developmental and epileptic encephalopathy 25 with amelogenesis imperfecta(615905)	ND	0.017	ND	↑	↓
SLC16A1 (MCT1)	Lactate, pyruvate	Erythrocyte lactate transporter defect(245340), hyperinsulinemic hypoglycemia(610021); monocarboxylate transporter-1 deficiency (MCT1D, 616095)	11.824	5.608	4.465	↓(0.032)	0.399
SLC16A2 (MCT8)	Thyroid hormones	Allan-Herndon-Dudley syndrome (AHDS, 300523)	1.870	0.911	0.565	↓(0.00024)	0.066
SLC19A3 (THTR2)	Thiamine	Thiamine metabolism dysfunction syndrome 2 (607483)	ND	0.218	0.167	↑	0.480
SLC25A1 (CIC)	citrate, isocitrate, malate, PEP	Combined D-2- and L-2-hydroxyglutaric aciduria(615182), Myasthenic syndrome, congenital, 23, presynaptic(618197)	4.035	3.960	4.166	0.840	0.686
SLC25A12 (AGC1)	L-Glu, D/L-Asp	Developmental and epileptic encephalopathy 39 (612949)	6.281	4.464	5.359	0.078	0.528
SLC25A13 (AGC2)	Aspartate, glutamate	Adult-onset type II Citrullinemia (CTLN2, 603471); Neonatal intrahepatic cholestasis caused by citrin deficiency (NICCD, 605814)	1.393	0.745	0.983	↓(0.00036)	0.355
SLC25A15 (ORC1)	Ornithine, citrulline	Hyperornithinemia-hyperammonemia-homocitrullinuria syndrome(238970)	0.055	0.044	ND	0.706	0.179
SLC25A19 (DNC)	Thiamine pyrophosphate	Microcephaly, Amish type(607196), Thiamine metabolism dysfunction syndrome 4(613710)	0.021	0.019	0.006	0.767	0.081
SLC25A20 (CAC)	Arnitine, acylcarnitine	Carnitine- acylcarnitine translocase deficiency (212138)	0.596	0.405	0.487	↓(0.025)	0.368
SLC25A22 (GC1)	L-Glu	Developmental and epileptic encephalopathy 3 (609304)	6.055	4.086	3.935	0.282	0.947
SLC25A3 (PHC)	Phosphate	Mitochondrial phosphate carrier deficiency (610773)	26.163	26.835	17.954	0.812	↓(0.0060)
SLC25A4 (ANT1)	ADP, ATP	progressive external ophthalmoplegia (PEO, 609283); mitochondrial DNA depletion syndrome (MTDPS, 617184; 615418)	5.837	10.034	5.230	↑(0.020)	↓(0.021)
SLC25A46		Neuropathy, hereditary motor and sensory, type VIB(616505), Pontocerebellar hypoplasia, type 1E(619303)	0.217	0.271	0.220	0.589	0.629
SLC26A2 (DTDST)	SO₄^2^⁻, oxalate, Cl-	Achondrogenesis Ib(600972), Atelosteogenesis, type II(256050)	0.024	ND	0.047	↓	↑
SLC27A4 (FATP4)	LCFA, VLCFA	Ichthyosis prematurity syndrome (608649)	0.380	0.395	0.517	0.890	0.332
SLC30A10 (ZnT10)	Zinc	Hypermanganesemia with dystonia-1 (613280)	0.167	0.078	ND	↓(0.012)	↓
SLC33A1 (ACATN1)	Acetyl-CoA	Spastic Paraplegia 42 (612539), Huppke-Brendel Syndrome (614482)	0.009	0.010	0.021	0.887	0.080
SLC37A4 (G6PT)	Glucose-6-phophate	Congenital disorder of glycosylation, type Iiw (619525), Glycogen-storage disease type Ib or Ic (232220 or 232240)	0.093	ND	0.092	↓	0.052
SLC40A1 (FPN1)	Ferrous iron	Hemochromatosis type 4 (606069)	0.104	0.078	ND	0.396	↓
SLC49A2** (FLVCR2)	Choline, heme	Proliferative vasculopathy and hydranencephaly-hydrocephaly syndrome (225790)	0.030	0.013	0.005	↓(0.035)	0.095
SLC52A2** (RFVT2)	Riboflavin	Brown-Vialetto-Van Laere syndrome (614707)	0.340	0.169	ND	↓(0.020)	↓
SLC52A3 (RFVT3)	Riboflavin	Brown-Vialetto-Van Laere syndrome (614707)	ND	ND	0.245	-	↑

*P values are calculated with Welch’s t-test. ** Detected with less than three razor + unique peptides.

ADP, Adenosine diphosphate; ATP, Adenosine triphosphate; CAA, cationic amino acid; LCFA, Long-chain fatty acids; NAA, neutral amino acid; PEP, phosphoenolpyruvate; VLCFA, Very-long-chain fatty acid. ND, not detected in more than 30% of samples in specific age group.

### ADME transporters are expressed on BBB

Due to limited transport across the BBB, most neurological drugs are lipid-soluble small molecules that rely on passive diffusion and must evade efflux by transporters like ABCB1 and ABCG2 to achieve therapeutic brain levels. Drugs unable to cross the BBB can be redesigned to use carrier- or receptor-mediated transport. SLC7A5 and TFRC which are well-known for their roles in endogenous BBB carrier-mediated and receptor-mediated drug transport respectively^27,28^, both exhibited a negative correlation with age. Their expression levels were highest in the Development group and decreased with age, showing reductions of 55.2% for SLC7A5 and 78.5% for TFRC compared to the Adult group (Supplemental Tables 2, 3).

**Fig. 4:**
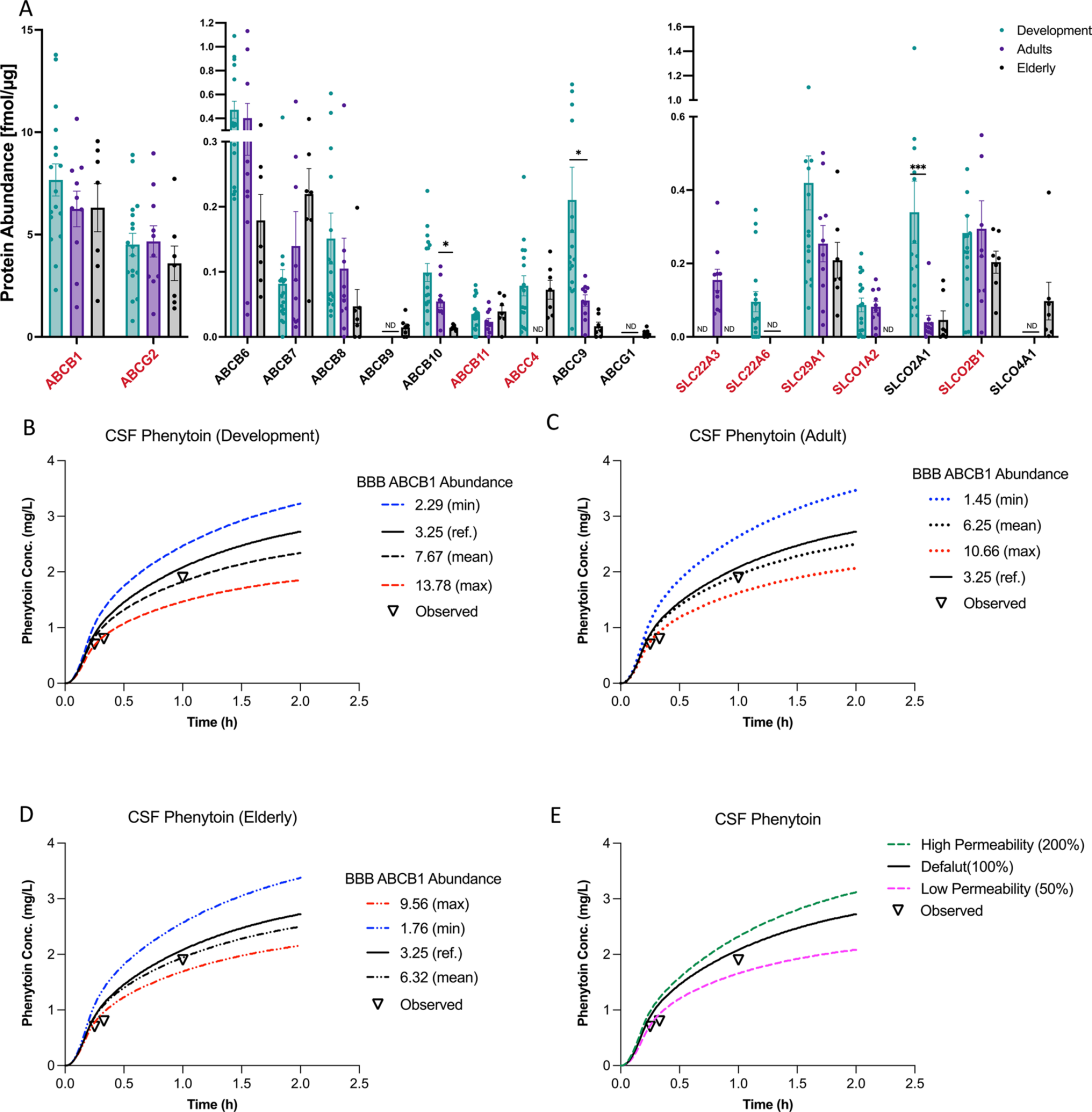
Changes in clinically important ADME transporters and BBB permeability potentially lead to different drug distribution in brain. (**A**) Clinically important uptake and efflux transporters (labeled red) and their family members in our BMV proteomic dataset are shown in bar graph. Kruskal–Wallis tests followed by Dunn’s post hoc test were used to assess differences between age groups in the bar graph. Data are represented as mean ± SEM and each point represents one sample. *P < 0.05, **P < 0.01, ***P < 0.001. ND, not detected in more than 30% of samples in specific age group. (**B**, **C**, **D**) Phenytoin time-concentration profile in spinal CSF with varying levels of P-gp (ABCB1) expression at the BBB. The default value from Simcyp is represented by the black solid line, the minimum value for each age group from BMV proteome is shown by the blue, and the maximum value from BMV proteome is indicated by the red. (**E**) Phenytoin time-concentration profile in spinal CSF with different BBB permeability. The default value is shown as the black solid line, 200% of the default BBB permeability is represented by the green dashed line, and 50% of the default BBB permeability is shown by the pink dashed line.

ABCB1 (P-gp), ABCG2 (BCRP), ABCC4 (MRP4), SLCO2B1 (OATP2B1), SLCO1A2 (OATP1A2), and SLC29A1 (ENT1) are clinically important transporters involved in the absorption, distribution, metabolism, and excretion (ADME) of drugs within the body^29^. These transporters, known to be expressed in the BBB^30^, were all identified in our BMV proteome (Fig. 4A). Consistent with prior studies, ABCB1 and ABCG2 were the most highly expressed among the ABC transporters. Age-dependent expression was evident in some transporters; ABCC4 is prevalent in Development and Elderly groups, while SLCO1A2 declined with age (Fig. 4A, Supplemental Fig. 6).

Other transporters that play a role in ADME in the BBB also exhibited differential expression with aging. SLC22A3 (OCT3) showed higher expression levels in Adult group but not in Development and Elderly groups. On the other hand, SLC22A6 (OAT1) is expressed only in early childhood (Fig. 4A). SLC22A8 (OAT3), which is highly expressed in the rodent BBB^31^, has been previously reported to be much lower or below the quantification limit in humans and monkeys^11,32,33^. Consistent with these findings, SLC22A8 was undetectable in our dataset, further highlighting species differences in BBB transport protein expression. Supplemental Table 4 provides a list of ABC and SLC transporters that play important roles in drug disposition, but do not appear to be expressed to a detectable degree in our BMV proteome.

### Simulations demonstrate the impact of transporter expression and BBB permeability on CSF drug exposure

As proof of concept, we employed physiologically based pharmacokinetic (PBPK) modeling and simulation with a published four-compartment, permeability-limited brain model to explore how individual differences in BBB passive permeability and ABCB1 expression affect the distribution of the anti-epileptic drug phenytoin to the CNS^34^. The simulations incorporated data on ABCB1 expression obtained from our proteomic analysis, as well as BBB permeability, to demonstrate the effects of variability in these parameters. The simulation results, depicted in Fig. 4B-E, demonstrate the concentration–time profiles of phenytoin in cerebrospinal fluid (CSF). Our simulations show that changes in ABCB1 expression and BBB permeability lead to potential clinical relevant changes (AUC changes > 1.25 fold or < 0.8 fold) in phenytoin exposure within the spinal cerebrospinal fluid (CSF) in each age group, with minimal impact on plasma phenytoin levels (Table 2, Fig. 4B-E, Supplemental Fig. 7). Additionally, lower BBB permeability results in a predicted decrease in phenytoin exposure in the CSF, for example, a two-fold reduction in BBB permeability results in a reduction in the CSF AUC by 21.0% (Fig. 4E, pink line). Conversely, higher BBB permeability results in a 12.3% increase in phenytoin CSF AUC (Fig. 4E, green line). Given the narrow therapeutic range of phenytoin, elevated CNS concentrations can lead to neurotoxicity, manifesting as symptoms ranging from mild nystagmus to ataxia, lethargy, and in severe cases, coma and death^35^. Interestingly, because of saturable metabolism, phenytoin has been associated with high interindividual differences in plasma levels^36^. Our findings suggest that, in addition to metabolic differences, the variable expression of ABCB1 in BMVs may further contribute to the large interindividual differences in both the efficacy and toxicity of phenytoin. Conventionally, serum phenytoin levels are monitored to ensure appropriate dosing and minimize adverse effects. However, our study suggests that serum phenytoin concentrations may not accurately indicate the risk for neurotoxicity, as they do not adequately reflect brain drug levels, which are attributable to differences in BBB permeability and transporter activity.

**Table 2:** Predicted effect of ABCB1 expression levels on phenytoin CSF exposure by Age Group.

Age group	ABCB1 expression (fmol/ug)		Fold Change to Ref* (3.25)	% Change in CSF AUC
Development	Max	13.78	4.24-fold ↑	–29.5
	Minimum	2.29	1.42-fold ↓	+ 18.6
Adult	Max	10.66	3.28-fold ↑	–21.9
	Minimum	1.45	1.85-fold ↓	+ 26.8
Elderly	Max	9.56	2.94-fold ↑	–18.7
	Minimum	1.76	1.85-fold ↓	+ 23.7

*Ref value is the default ABCB1 expression level from Simcyp.

### Proteins associated with higher risk of AD are correlated with age and differentially expressed in AD patients

**Fig. 5:**
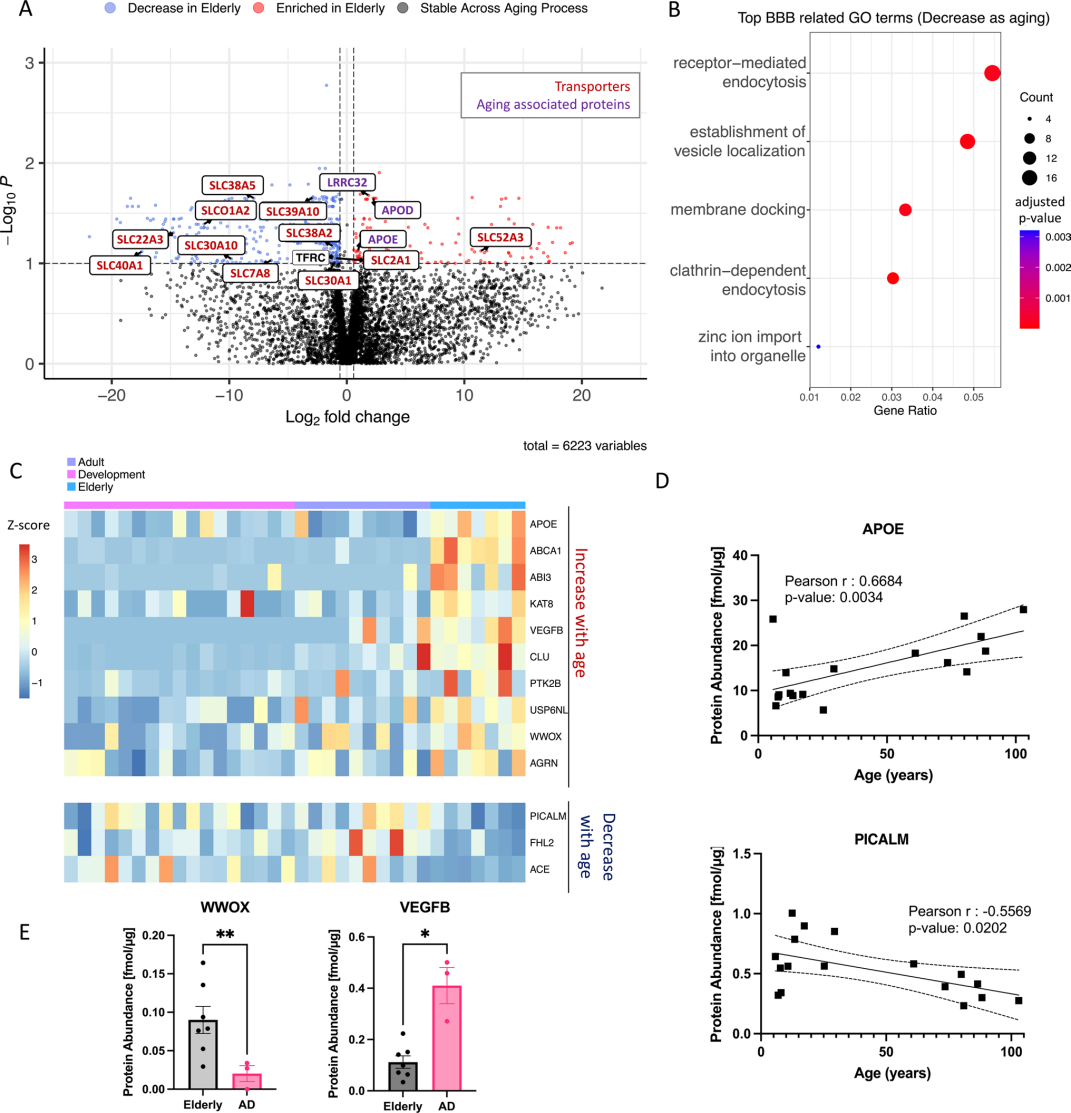
A subset of proteins associated with a higher risk of AD shows age-related expression patterns and are differentially expressed in AD patients. (**A**) Volcano plot displaying all proteins differentially expressed between Adult group (N = 10) and Elderly group (N = 7) of the BMV proteome. Proteins with significantly decreased levels in elderly population (*P* < 0.1) are labeled in blue, while the proteins with significantly increased levels with aging are shown in red. Transporters are labeled in red and proteins known to play crucial roles in aging are labeled in purple. (**B**)Top BBB related GO terms associated with proteins significantly decreased with age are shown. X-axis is the gene enrichment ratio (Gene ratio) and the bubble size indicates the number of proteins associated a biological process GO term, with color mapping the FDR value (adjusted *p-*value) of the enrichment analysis. (**C**) Protein expression of AD GWAS genes which are expressed in our proteomic dataset and exhibit positive(red) or negative(blue) correlation with age. Scale represents the row Z-score (each row presents one protein), which is calculated by taking each individual’s protein expression, subtracting the mean expression, and then dividing by the standard deviation of that protein. (**D**) Protein abundance of APOE and PICALM through aging are described as simple linear regression model. Individual curves are presented in (solid lines). Dashed lines represent the 95% confidence bands. (**E**) Protein abundance levels of WWOX and VEGFB in healthy elderly population or in patients with AD. Expression differences between disease condition were assessed by student T test. *P < 0.05, **P < 0.01, ***P < 0.001.

AD presents a significant challenge in healthcare due to the lack of effective curative treatments. Since AD is closely associated with aging, this section specifically focuses on understanding the aging process of the BBB. Differential protein expression between the Adult (N = 10) and Elderly groups (N = 7) revealed a total of 140 proteins with significantly increased abundance and 357 proteins with significantly decreased abundance during the aging process (Fig. 5A). These differentially expressed proteins include key aging-related proteins, such as Apolipoprotein D and LRRC32, involved in TGF-beta regulation. GO enrichment analysis of these 140 upregulated proteins showed strong associations with *cell adhesion* and *leukocyte migration* (Supplemental Fig. 4B). Conversely, the 357 proteins with significantly decreased expression were strongly associated with *endocytosis*, *cell junction maintenance*, and *zinc ion transmembrane transport* (Fig. 5B).

To explore whether proteins that change expression during aging might be linked to AD, we examined 45 AD-related genes identified from Genome-Wide Association Studies (GWAS)^7^ within our BMV proteome. Of these, 22 were detected, and 12 showed age-related expression patterns(Fig. 5C); eight were significantly correlated with aging, including one protein only present in the elderly group (Fig. 5D, Supplemental Fig. 7). WW domain-containing oxidoreductase (WWOX) an AD risk factor involved in neurodegeneration, was reduced in AD patients compared to healthy elderly controls^37^(Fig. 5E). Furthermore, aging is acknowledged as a principal risk factor for AD; however, the aging process of BMVs is still largely unknown, particularly with regard to its link to AD risk. In our BMV dataset, eight AD GWAS genes demonstrate age-specific correlations, underscoring their potential contribution to the role of aging in AD. Apolipoprotein E (APOE)—critical in AD pathology—showed higher expression with age. Conversely, the phosphatidylinositol binding clathrin assembly protein (PICALM), one of the most significant loci identified in AD susceptibility GWAS, displayed a negative correlation with aging. Furthermore, vascular endothelial growth factor B (VEGFB), important for BBB repair, increased with age and was further elevated in AD patients, aligning with its association with BBB disruption and cognitive decline.

## Discussion

This study aimed to assess how development and aging affect BBB, focusing on transporter expression relevant to CNS drug disposition and nutrient delivery. Our key findings include: (a) Alterations in basement membrane components and increased expression of cell adhesion molecules occur during early development; (b) Several brain-essential nutrient transporters are enriched in early development; (c) Certain drug transporters at the BBB show age-dependent expression; (d) Exploratory data indicate age-dependent expression of some AD-related proteins in BMVs. Each finding is discussed below, followed by study limitations.

### Alterations in basement membrane components and increased expression of cell adhesion molecules occur during early development.

Unlike the cellular components of the BBB, the basement membrane has only been recently recognized for its importance in maintaining BBB permeability^1^. Age-related changes were evident in the rodent BBB’s basement membrane^38^. Our BBB proteome study, for the first time, comprehensively identified the collagen families composing the basement membrane in human across various age groups (from neonates to the elderly). In early childhood, the Collagen VI family (COL6A1 and COL6A3) is the most highly expressed, whereas in adulthood, the Collagen IV family (COL4A1 and COL4A2) is predominant. These two collagens are distinct in terms of their morphology. Collagen VI is an unusual collagen comprising both collagenous and non-collagenous domains that assemble into beaded filaments, whereas Collagen IV forms a protomer fiber made of three α chains. Collagen IV is well-known to form the backbone of the basement membrane^39^. The Collagen VI family is highly expressed in various human tumors and influences cell proliferation and angiogenesis^40^. Since it is highly expressed in neonates, this aligns with the need for postnatal vascularization at the early developmental stage. We have also observed increased perlecan expression with age in our dataset. Beyond its structural role, perlecan and its cleavage products have been shown to participate in BBB protection and neurorepair. In particular, studies from Bix and colleagues demonstrated that perlecan domain V promotes angiogenesis, neuronal survival, and BBB stabilization following ischemic injury, highlighting its neuroprotective potential^41^. These findings raise the possibility that age dependent increase of perlecan expression represents an adaptive response that enhances vascular integrity and BBB resilience, rather than merely reflecting extracellular matrix turnover.

Cell adhesion molecules (CAMs) at the BBB mediate cell–cell and cell–matrix interactions. Our study showed that major junctional proteins (TJP1, CLDN5, OCLN, CDH5) remain stable throughout life, while CLDN11, although much lower in abundance than CLDN5, significantly increases with age (Supplemental Fig. 1). CLDN11 knockdown increases endothelial permeability, suggesting a role in BBB integrity, highlighting the potential functional relevance of these interactions in human BBB development. Integrin β1 (ITGB1), the most abundant integrin in our BMV proteome, rises during maturation, peaking in adulthood before declining with age. ITGB1 supports endothelial sprouting in development and maintains VE-cadherin localization, crucial for junction integrity in mature vessels^42^. Its increased expression in adults likely supports early postnatal vascularization and later BBB maintenance. Further research is needed to clarify how different collagens and age-related changes in junctional proteins and integrins influence BBB permeability.

### Transporters important for CNS nutrient disposition and brain development are mainly enriched during early development

Our study is the first to comprehensively map nutrient transporter expression at the human BBB across development and aging. Prior rodent studies have reported higher expression of Slc1a4, Slc7a1, and Slc38a5 at the BBB during the neonatal period, and elevated Slc16a1 expression in early developmental stages has also been observed in both rodents and cynomolgus monkeys^38,43–45^. Our present study in humans further supports this early need for high concentration of nutrients in the brain with higher expression of SLC1A4, SLC7A1, SLC16A1 and SLC38A5 (SNAT5) in early childhood. This also aligns with clinical findings that these amino acids present higher CSF to plasma concentration ratios in infancy relative to later in childhood^46^. Moreover, while no metabolic studies have focused on transporter-specific metabolites in aging, several have shown age-related changes in CSF metabolites linked to amino acid metabolism and redox processes, suggesting increased oxidative stress in the aging brain^47,48^. Our WGCNA analysis identified a module (M27) linked to ROS biosynthesis that was significantly upregulated in the elderly, including CYBA (a NOX2 subunit) and the oxidative stress marker LCN2.

Compared to previous human BBB global proteomic studies^13^, our inclusion of developmental samples allowed us to identify several important nutrient transporters at early stages, including SLC52A2 (riboflavin transporter) and SLC49A2 (heme/choline transporter), as well as SLC5A6, SLC19A3, and SLC52A3, which were not previously reported.

### ADME transporters on BBB exhibit age-dependent patterns

Many known clinically important ADME transporters, including ABCB1, ABCG2, ABCC4, SLCO2B1, SLCO1A2, and SLC29A1, are expressed in our BMV proteome^30^. Consistent with previous studies, ABCB1 and ABCG2 were the most abundant. While no significant age-related changes were observed, we found substantial interindividual variability in their expression, ranging from 1.45 fmol/µg to 13.78 fmol/µg for ABCB1, and from 0.78 to 8.97 fmol/µg for ABCG2. This large variation could potentially impact brain drug exposure and toxicity. Age-dependent variation in expression was observed for some ADME transporters such as SLCO1A2, ABCC4, and SLC22A6. Altered expression of these ADME transporters may not only affect drug efficacy but also contribute to toxicity^49^. Methotrexate (MTX) is linked to neurotoxicity, with younger patients showing lower risk than older ones^49,50^. Age-dependent changes in transporters related to brain penetration of MTX including OAT1 and ABCC4 may contribute to this difference in susceptibility. (Supplemental Fig. 6). Our proteomic analysis further confirmed the species differences in BBB transporter expression, emphasizing the importance of human-based BBB studies for improving CNS drug development. Consistent with previous reports^32,33,43,51^, our data showed that SLC22A8 (OAT3) expression is undetectable in human brain microvessels, whereas SLCO2B1 is the predominantly expressed SLCO transporter in the adult human BBB. In contrast, SLCO2A1 exhibited the highest expression among SLCO family members during early development. These findings emphasize the need to account for species- and developmental stage–specific differences in transporter expression when extrapolating BBB permeability and drug disposition from animal models to humans^52^.

### Age is a significant risk factor for many neurological disorders including AD

In our study using BMV samples from elderly patients, we discovered that 8.6% (595) of proteins change with aging. Proteins involved in inflammation increase with age, while zinc transporters decline—potentially linking zinc deficiency to higher AD risk through inflammation. Our findings reveal a reduction in the expression of zinc transporters SLC30A1, SLC30A5, and SLC30A10 among the elderly. In AD, the BBB is disrupted by increased permeability of BMVs through the downregulation of junction proteins^53,54^. In response to BBB damage, VEGFB, a key factor in BBB repair, has been shown to be upregulated in AD patients^55^, consistent with our observations of higher VEGFB levels in AD within our dataset. Our study also reveals for the first time that VEGFB is absent in early childhood and significantly increases with age. Although further validation with larger sample sizes is needed, our data provide additional evidence that BBB permeability and function should be key considerations in AD drug development.

The major limitations of this study include the following. First, there is contamination of other brain cell types in our BMV samples, however, this type of potential contamination has been reported in other studies when characterizing the BBB (Supplemental Table 5^12,13^,). To further evaluate how the variations in cellular contamination might contribute to age-related changes in our dataset, we first confirmed the abundance of the proteins in each cell type based on publicly available single-cell RNA-seq datasets (Brain-Seq, https://brainrnaseq.org/), then assessed whether the expression of age-related transporter proteins correlated with astrocyte marker(GFAP) and pericyte marker (CSPG4) and whether the direction of correlation was consistent with the trends observed in our study (Supplementary Tables 6 and 7). No detectable levels of IBA1 or TMEM119, two well-established microglial markers, indicating minimal microglial presence in our samples. Despite potential contamination, BMV-specific markers were enriched, and no age correlation was found with CDH5 and PECAM1, supporting effective BMV isolation (Supplemental Figs. 8, 9). Previous studies have been done to assess transporter proteins at the BBB; however, they’ve been limited to adult brains^11–13^. Consistent with prior studies, we found SLC2A1 as a top SLC and ABCB1 and ABCG2 as the most abundant ABC transporters at the BBB. However, our BMV proteome analysis also revealed values inconsistent with those found in other studies. For instance, we found consistent CLDN5 expression across ages, differ from prior reports of developmental increases, possibly due to our broad age ranges and limited infant samples (Wolburg et al., 2004). Tight junctions such as CLDN5, OCLN, and TJP1 form during fetal development and continue to mature postnatally through increased structural complexity and regulatory refinement. However, changes occurring during birth and early infancy (0–180 days) may be subtle and difficult to detect in bulk proteomic analyses, particularly given the broad age range of samples. Denser sampling within the first year could better capture these early developmental dynamics. An additional limitation of our study is that brain samples were obtained from the University of Maryland NeuroBioBank, although no significant correlation was observed between CDH5 level and postmortem intervals (PMI, Supplementary Fig. 9), variability in PMI could still have influenced the stability of certain proteins. Finally, in the Elderly group, samples were from a small number of donors, especially for the samples from AD patients (N = 3). Studies with larger sample sizes are needed to confirm all of these results.

In conclusion, our study mapped age-dependent expression of key BMV proteins, revealing physiological brain changes across the lifespan and potential mechanisms behind age-related differences in drug distribution, metabolism, and CNS sensitivity. Using a phenytoin PBPK model, we demonstrated how transporter variation affects brain drug exposure, emphasizing the need to consider BBB dynamics in dosing guidelines. Building on this proof-of-concept analysis, future studies, with support of clinical PK and transporter expression data, should extend the modeling framework to include BCRP and dual P-gp/BCRP substrates, as well as key SLC transporters to capture the interplay between influx and efflux mechanisms at the BBB. Such efforts will enable a more comprehensive understanding of transporter-mediated brain drug disposition. Our findings support integrating age-related BBB data into CNS drug development to improve safety and efficacy, especially for pediatric and elderly patients, ultimately advancing personalized, age-tailored CNS therapies.

## Methods

### Study design and human brain tissue samples

Post-mortem frozen brain cortical tissue samples from thirty-four healthy donors and three Alzheimer’s Disease patients were obtained from the National Institutes of Health NeuroBioBank at the University of Maryland, Baltimore, MD. The brain tissues obtained from NeuroBiobank are not considered as human subjects per U.S. Department of Health and Human Services (DHHS) definition and the tissues obtained were de-identified and contained no Protected Health Information. Tissues were stored at − 80 °C until the day of microvessel isolation. The demographic information of donors is reported in Supplemental Table 1. The pediatric population was separated into different age groups according to the International Council for Harmonisation guidelines: newborns (PNA 0–28 days, GA > 37 weeks), infants (1–24 months old), children (2–12 years old), and adolescents (12–16 years old). Donors over 16 years old were defined as adults and donors over 60 years old were defined as Elderly group. However, due to small sample sizes, newborns and infants were combined into “Development group” and children, adolescents, and adults were combined into “Adult Group” to increase the power of the study. We conducted a differential protein expression analysis to compare the neonate group (0 to 28 days) with the infant group (1–6 months), as well as the children group (4–12 years) with the adult group (12–30 years). According to our significance criteria (absolute log2 fold change > log2(1.5), *P* value < 0.1), no significantly differentially expressed proteins were identified in these comparisons (Supplementary Fig. 10). LC–MS/MS protocols were approved by Uppsala University, and all other experimental procedures were approved by the University of California, San Francisco. All methods were carried out in accordance with relevant guidelines and regulations.

### Isolation of human brain microvessels

The process of isolating brain microvessels (BMVs) from human brain cortical tissue was conducted with some modifications based on a previously described protocol^56^. The procedure started with using less than 1 g of tissue which was thawed and homogenized in protease inhibitors (cOmplete protease inhibitor cocktail, Sigma-Aldrich) contained Hanks’ Balanced Salt Solution(HBSS, Thermo Fisher) with 20 up- and-down strokes in a Potter–Elvehjem glass homogenizer. The resulting homogenate was centrifuged at 1200 g for 10 min at 4 °C, and the BMV enriched pellet was resuspended in a 17.5% dextran-70/HBSS solution and centrifuged at 4300 g for 15 min at 4 °C in a swinging bucket rotor. The myelin-rich layer on the top and the supernatant were aspirated and the pellet was resuspended in HBSS with 1% Bovine Serum Albumin (BSA, Sigma-Aldrich). This solution was filtered with a 40 μm nylon mesh strainer and the BMVs captured on the strainer were washed with 35 ml of 1% BSA/ HBSS buffer. The BMVs were then collected off the filter with 1% BSA/HBSS buffer and centrifuged at 3000 g for 5 min at 4 °C. The supernatant was removed and the resulting BMV pellet was immediately frozen at -80 °C for further analysis. All the steps were carried out at 4 °C or on ice. Quantitative Polymerase Chain Reaction (qPCR) was performed to assess the enrichment of brain microvessels in our samples by measuring the relative expression (RQ values) of endothelial, neuronal, and astrocyte markers. The RQ values of the following proteins, which are considered endothelial cell type biomarkers: GLUT1, LAT1 and ABCB1 were enriched 8.68, 33.3 and 54.9 -fold in our brain microvessel preparation compared to the original brain homogenate. SYP, a biomarker for neurons was < 0.1 of the original homogenate in our microvessel samples, consistent with diluting neurons in our preparations and no enrichment of GFAP, the astrocyte biomarker, was observed in our preparations.

### Global proteomics using liquid chromatography tandem mass spectrometry (LC–MS/MS)

The process of LC–MS/MS is based on a previously described protocol^56^. BMV samples were lysed in a 100 mM Tris–HCl buffer (pH 7.8) containing 50 mM dithiothreitol and 2% sodium dodecyl sulfate, followed by a 5-min incubation at 95 °C. Subsequently, the samples underwent sonication using a Branson-rod-type sonicator and were then centrifuged at 14,000 g for 10 min. To determine protein concentration, a tryptophan fluorescence assay was employed. For multi-enzyme digestion, 100 μg of protein was utilized in the multi-enzyme digestion filter-aided sample preparation (MED-FASP) protocol, where sequential digestion with LysC and trypsin occurred. Following digestion, the resulting peptides were concentrated via a GeneVac EX-2plus system. Subsequently, peptide separation was achieved using an Ultimate 3000 RSLCnano system, employing an easy spray C18 reversed-phase column (50 cm, ID 75 μm) with a gradient of water/acetonitrile containing 0.1% formic acid over a 145 min period. The eluted peptides were analyzed with a Top15 method, involving full MS followed by ddMS2 scans, using an Orbitrap Q Exactive HF mass spectrometer (ThermoFisher, Waltham, MA). Data analysis was conducted using MaxQuant version 1.6.10.43, with the complete human proteome extracted from UniProt (September 2020). A false discovery rate of 0.01 was set, and match-between-runs was enabled for enhanced accuracy. Protein abundance quantification was performed using the total protein approach (TPA)^57^ for proteins with three or more razor + unique peptides. Briefly, protein concentration was calculated as follows:

Protein (i) = MS signal (i) / Total MS signal x MW (i) [pmol/mg total protein].

The mass spectrometry proteomics data have been deposited in the ProteomeXchange Consortium through the PRIDE partner repository. In total, we identified 9005 proteins across all samples. To address missing data and proteins expressed only in specific age groups, we only selected proteins detected in at least 70% of samples within one or more age groups. This filtering resulted in 6,887 proteins. Of these, 6,223 proteins were considered as quantifiable (detected with three or more razor + unique peptides) and subsequently used for expression analysis.

### Weighted gene correlation network analysis of discovery brain proteome

Weighted protein co-expression network of the discovery BMV dataset was derived from the protein abundance values using the blockwiseModules WGCNA function^19^ (WGCNA 1.72.5 R package) with the following settings: soft threshold power beta = 6, deepSplit = 2, minimum module size = 30, merge cut height = 0.25, TOMDenominator = “min”, a signed network with partitioning about medioids (PAM) respecting the dendrogram. This approach calculates pairwise biweight mid-correlations (bicor, a robust correlation metric) between each protein pair and transforms this correlation matrix into a signed adjacency matrix. After blockwiseModules network construction, 30 modules consisting of 34 or more proteins were detected. Module eigenproteins, which are defined as the first principal component (PC) of each module protein expression, were correlated with age(days) using bicor analysis. We identified the most significantly GO term associated with each module using Cytoscape. Specifically, we utilized the INDRA GO analysis function within the NDEx Integrated Query tool in Cytoscape^20,58^. This tool uses the INDRA system to integrate outputs from multiple automated literature-mining sources, assembling pathway knowledge to generate networks of interactions and regulations for genes annotated with a given GO term.

### Gene ontology enrichment analysis

The enriched GO terms (biological process, cellular component, and molecular function) of the differentially expressed proteins during the developmental stage and aging process were identified and visualized based on the clusterProfiler version 4.4.4 package in R software using the default settings^59^.

### Physiologically based pharmacokinetic (PBPK) modeling

Systemic and central nervous system (CNS) drug concentrations were simulated using both whole-body and four-compartment permeability-limited brain models within the Simcyp PBPK Simulator (version 21.1 and 22, Certara, Princeton, NJ, USA). The pharmacological and physiological parameters for the model were adapted from a previously published PBPK model for phenytoin^34^. Simulations were conducted over 10 trials, each consisting of 10 virtual healthy volunteers, using the same demographic and dosing information as the referenced phenytoin model. Default settings for BBB ABCB1(P-gp) abundance (3.25 pmol/mg of brain capillary protein) and permeability (46.44 L/h) were applied to generate baseline curves. To mirror the proteomic data observed in our study, adjustments were made to these parameters, and the results of these modifications are presented with the respective parameter values in Fig. 4B-E.

### Statistical analysis

For differential protein expression analysis where we compare the protein expression differences between Development group and Elder group with Adult group respectively, unpairwise differentially expressed proteins were identified using Student’s t-test, followed by Benjamini-Hochberg (BH) FDR correction (significance defined as Absolute Log2 Fold Change > Log2(1.5), *P* value < 0.1). Differential expression was presented in volcano plots, which were generated with the “EnhancedVolcano” version 1.14.0 package in R^60^. When comparing the significant differences in protein expression across three age groups, we performed Kruskal–Wallis tests followed by Dunn’s post hoc test were used to compare the mean of each age groups with the mean of the Adult group. Data are represented as mean ± SEM and each points represent one sample. Statistical significance was indicated as *P < 0.05, **P < 0.01, ***P < 0.001 and if certain proteins was not detected in more than 30% of samples in specific age group, we defined this protein as non-detected in our analysis based on the filtration criteria referred in result section. For specific differences and more detailed information, please refer to the figure legend.

## Supplementary Information

Below is the link to the electronic supplementary material.

Supplementary Material 1

Supplementary Material 2

Supplementary Material 3

Supplementary Material 4

## Data Availability

The mass spectrometry proteomics data that support the findings of this study have been deposited in the ProteomeXchange Consortium through the PRIDE partner repository with the project accession codes PXD057192 and full access are available from the corresponding author upon reasonable request. The authors declare that all the processed data supporting the findings of this study are available within the paper in Supplementary_Data_2.

## Code availability

The code used for analysis in this study has been deposited in Zenodo [10.5281/zenodo.15732703] and available on GitHub [https://github.com/xujia0525/BBB_proteomic_analysis].
