# Supplementary material for: Proteomic profiling reveals age-related changes in transporter proteins in the human blood–brain barrier
Source: Sci Rep. 2025 Dec 26;16:1698. doi: 10.1038/s41598-025-31224-6 (PMC12800244; doi:10.1038/s41598-025-31224-6)
Supplement: Supplementary file 4 — Supplementary Material 4 [file 41598_2025_31224_MOESM4_ESM.pdf]

Supplemental Figures

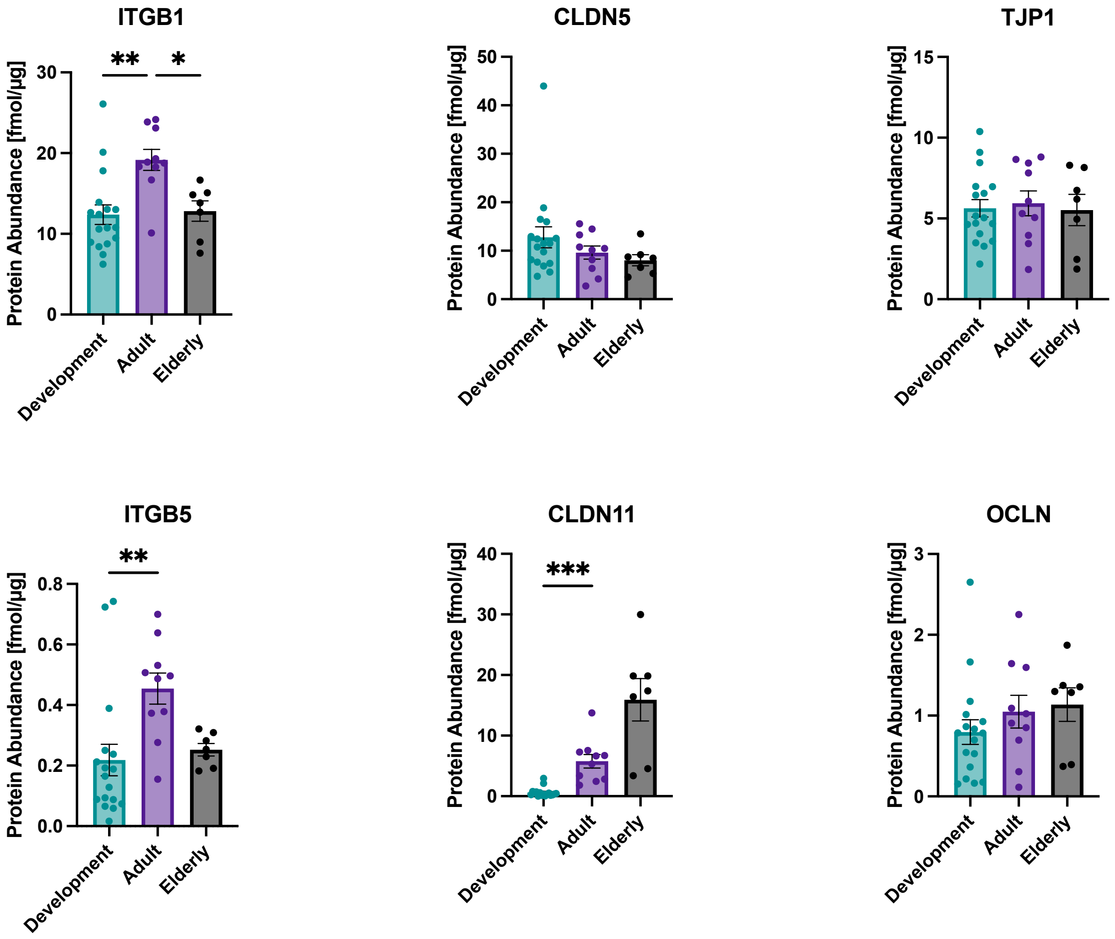

Supplemental Figure 1. Major tight junction proteins and integrins on BBB
Representative tight junction proteins and adhesion molecules in our BMV proteomic dataset are shown in bar graph, bars represented the mean of the transporter expressions in different age groups. For the bar graphs Kruskal–Wallis tests followed by Dunn's post hoc test were used to compare the mean of each age groups with the mean of the Adult group. Data are represented as mean ± SEM and each points represent one sample. *$P < 0.05$, **$P < 0.01$, ***$P < 0.001$. ND, not detected in more than 30% of samples in specific age group.

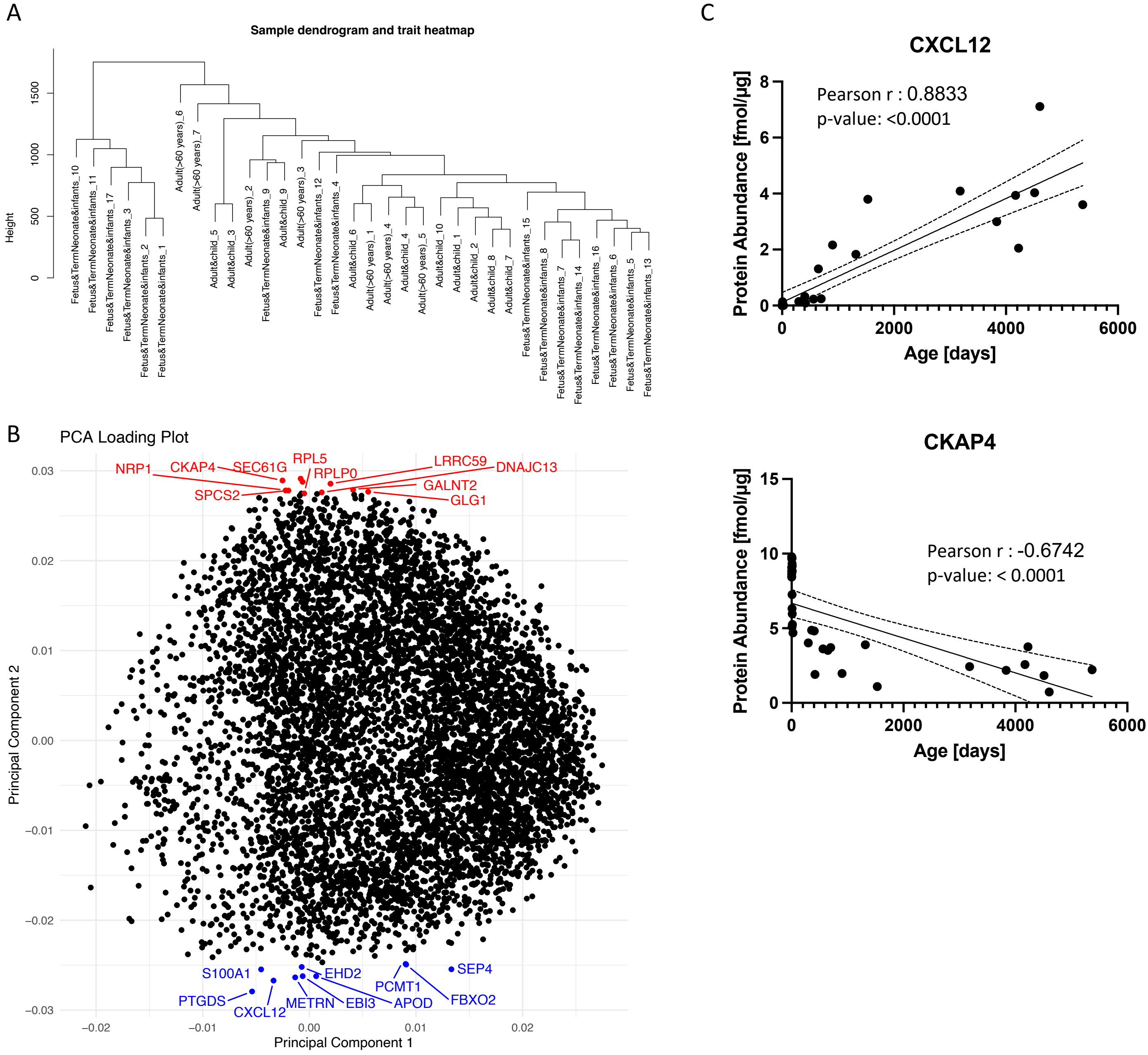

Supplemental Figure 2. PCA loadings plot on BMV proteomic dataset and proteins related to the observed clustering

(A) Sample clustering dendrogram of our BMV proteome to detect outliers. (B) Principal component analysis (PCA) loadings plot on BMV proteomic dataset. The top 10 proteins that positively or negatively influence PC2 are labeled and shown in red (positive) or blue (negative), respectively. (C) Ontogeny of protein abundance of CKAP4 and CXCL12 are described as simple linear regression model. Individual curve are presented in (solid lines). Dashed lines represent the 95% confidence bands. Individual Pearson correlation coefficient (Pearson r) and p-values are presented in the plots.

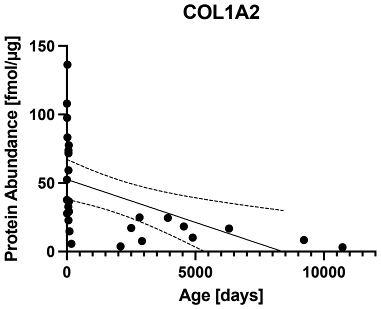

Pearson r : -0.523
p-value: 0.0051

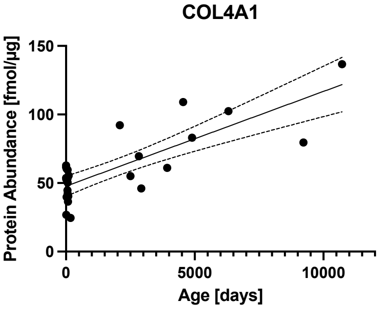

Pearson r : 0.800
p-value: < 0.0001

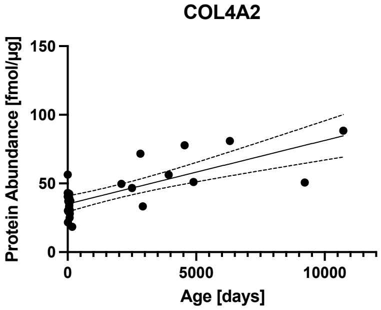

Pearson r : 0.755
p-value: < 0.0001

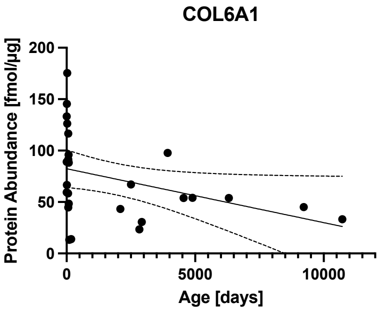

Pearson r : -0.381
p-value: 0.0501

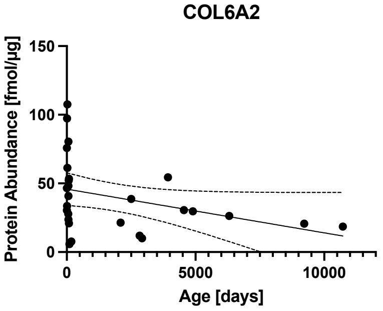

Pearson r : 0.359
p-value: 0.0663

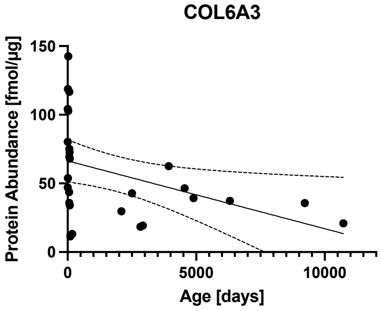

Pearson r : -0.419
p-value: 0.0294

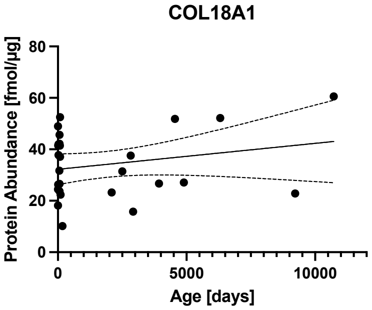

Pearson r : 0.234
p-value: 0.239

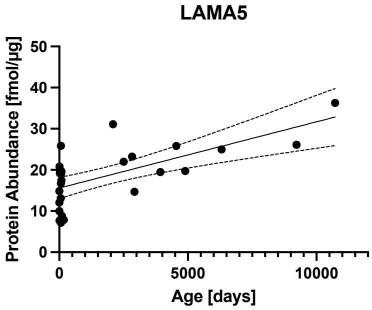

Pearson r : 0.665
p-value: 0.00016

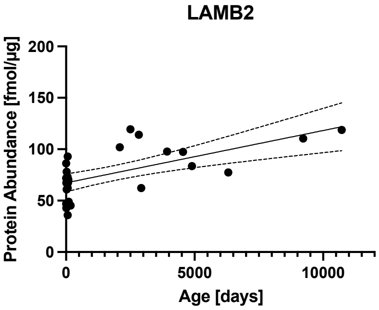

Pearson r : 0.643
p-value: 0.0003

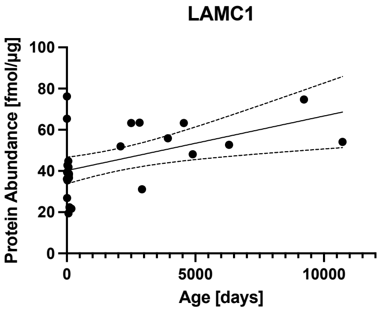

Pearson r : 0.507
p-value: 0.00694

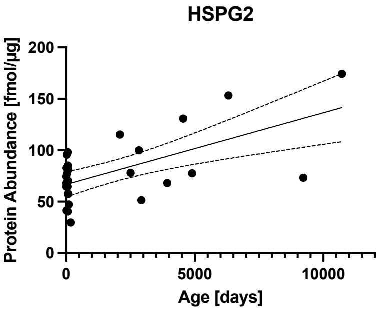

Pearson r : 0.628
p-value: 0.00046

Supplemental Figure 3. Basement Membrane Components Alters During Developmental Stage
Ontogeny of protein abundance of representative collagens, laminins and perlecan are described as simple linear regression model. Individual curve are presented in (solid lines). Dashed lines represent the 95% confidence bands. Individual Pearson correlation coefficient (Pearson r) and p-values are presented under the plots.

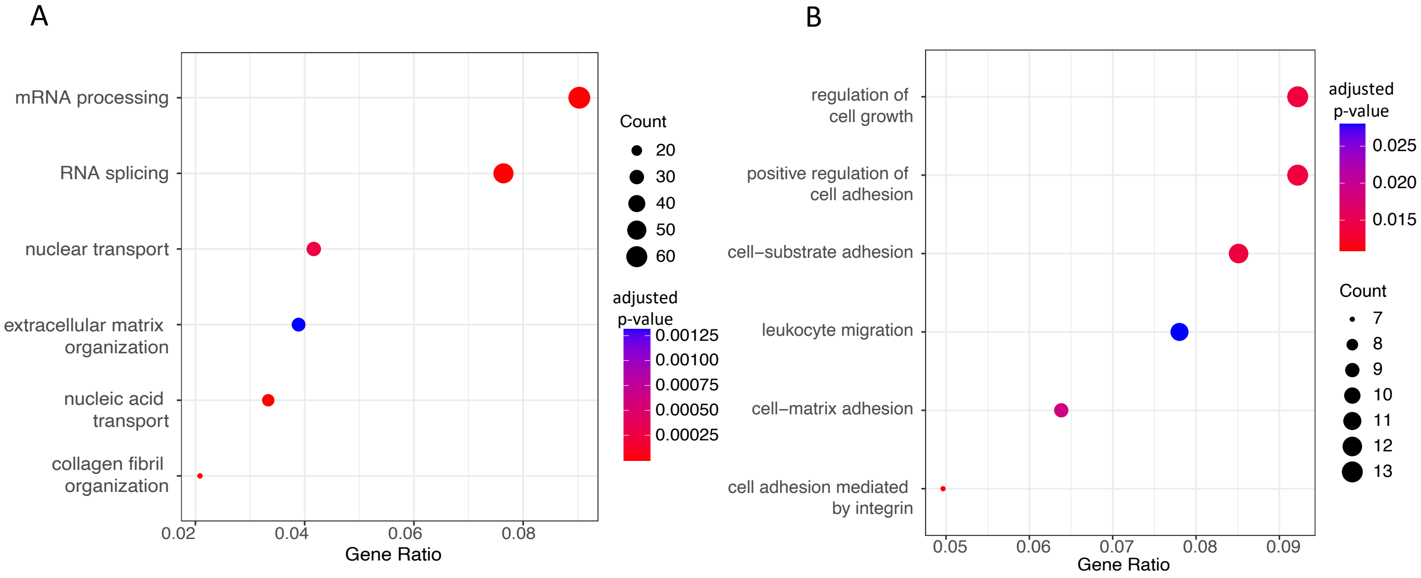

Supplemental Figure 4. GO enrichment analysis of proteins changing with age
(A) GO terms associated with proteins significantly enriched in Development group are shown. X-axis is the gene enrichment ratio (Generatio) and the bubble size indicates the numbers of proteins associated a biological process GO term, with color maps the FDR value (adjusted p-value) of the enrichment analysis. (B) Top BBB related GO terms associated with proteins significantly increased through aging process are shown. X-axis is the gene enrichment ratio (Generatio) and the bubble size indicates the numbers of proteins associated a biological process GO term, with color maps the FDR value (adjusted p-value) of the enrichment analysis.

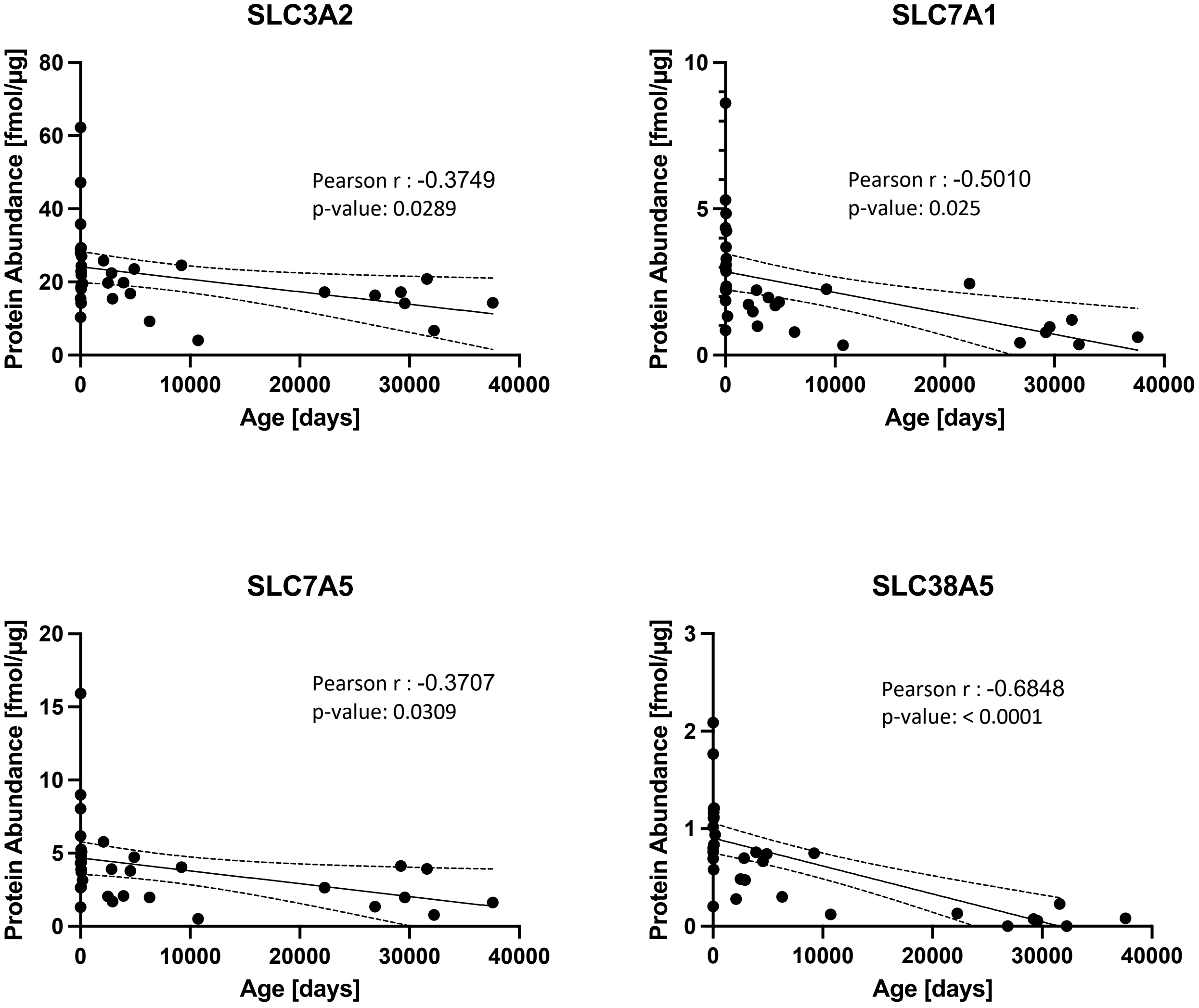

Supplemental Figure 5. Amino Acid Transporter are Enriched in Early Childhood

Ontogeny of protein abundance of amino acid transporters SLC3A2, SLC7A1, SLC7A5, SLC38A5 are described as simple linear regression model. Individual curve are presented in (solid lines). Dashed lines represent the 95% confidence bands. Individual Pearson correlation coefficient (Pearson r) and p-values are presented in the plots.

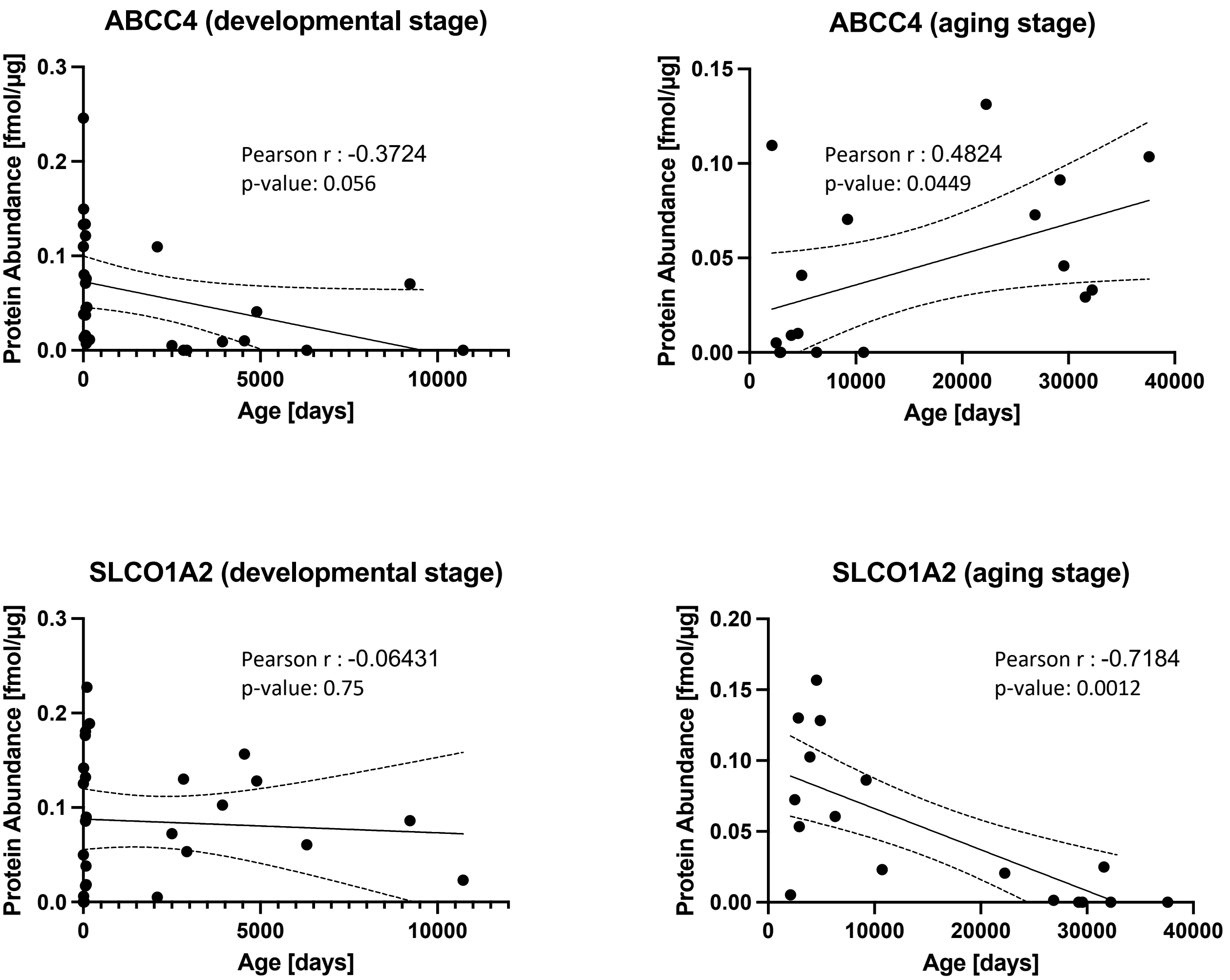

Supplemental Figure 6. Correlation plot with age for clinically important ADME transporters

Ontogeny of protein abundance of clinically important ADME transporters ABCC4 and SLCO1A2 are described as simple linear regression model (A,B developmental stage; C,D aging stage). Individual curve are presented in (solid lines). Dashed lines represent the 95% confidence bands. Individual Pearson correlation coefficient (Pearson r) and p-values are presented in the plots.

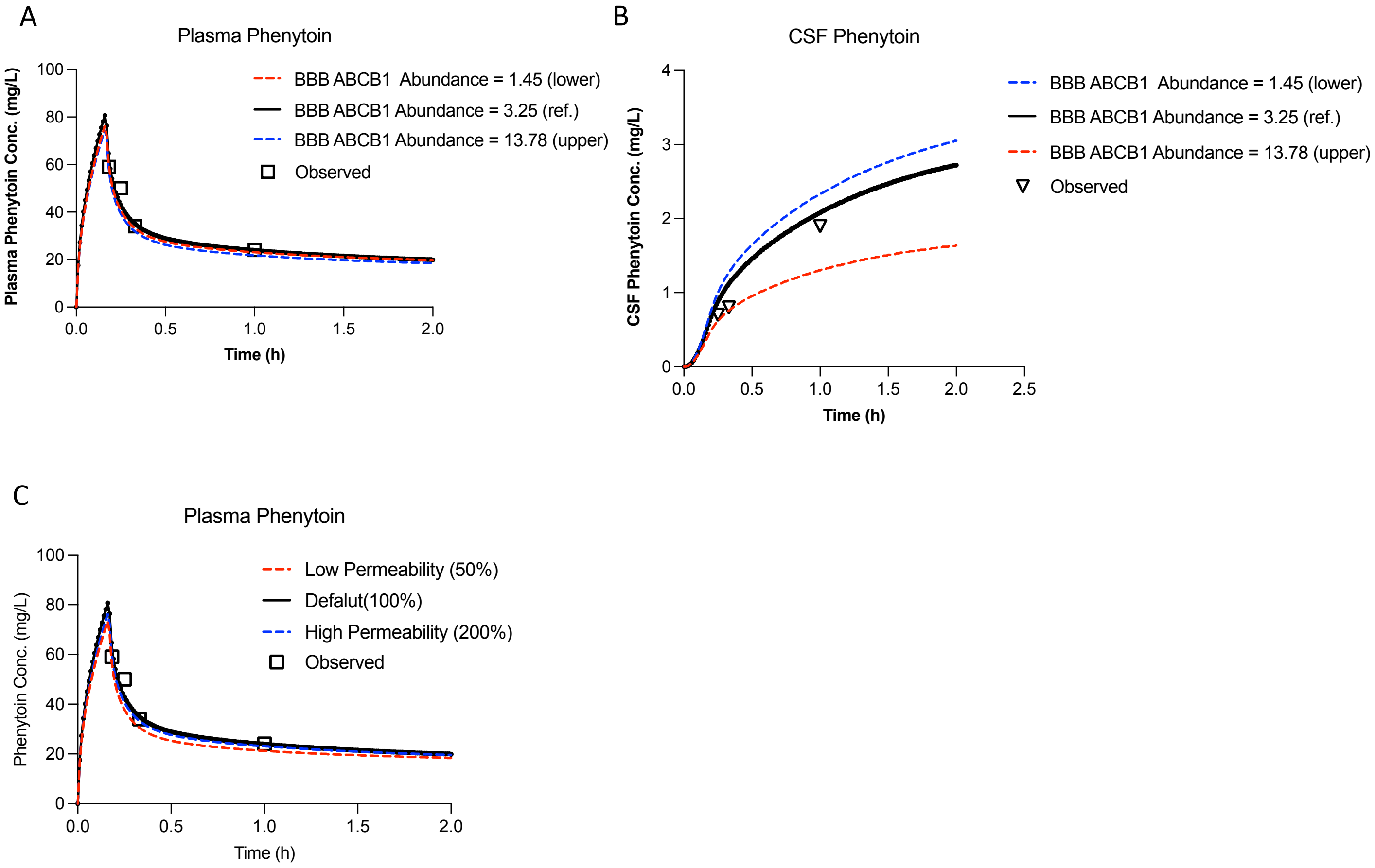

Supplemental Figure 7. Changes in BBB ABCB1 level and BBB permeability lead to no difference in plasma drug distribution.

(A, B) Phenytoin time-concentration profile in plasma and CSF with varying levels of P-gp (ABCB1) expression at the BBB. The default value is represented by the black solid line, the minimum value from BMV proteome is shown by the blue dashed line, and the maximum value from BMV proteome is indicated by the red dashed line.(C) Phenytoin time-concentration profile in plasma with different BBB permeability. The default value is shown by the black solid line, 200% of the default BBB permeability is represented by the blue dashed line, and 50% of the default BBB permeability is shown by the red dashed line.

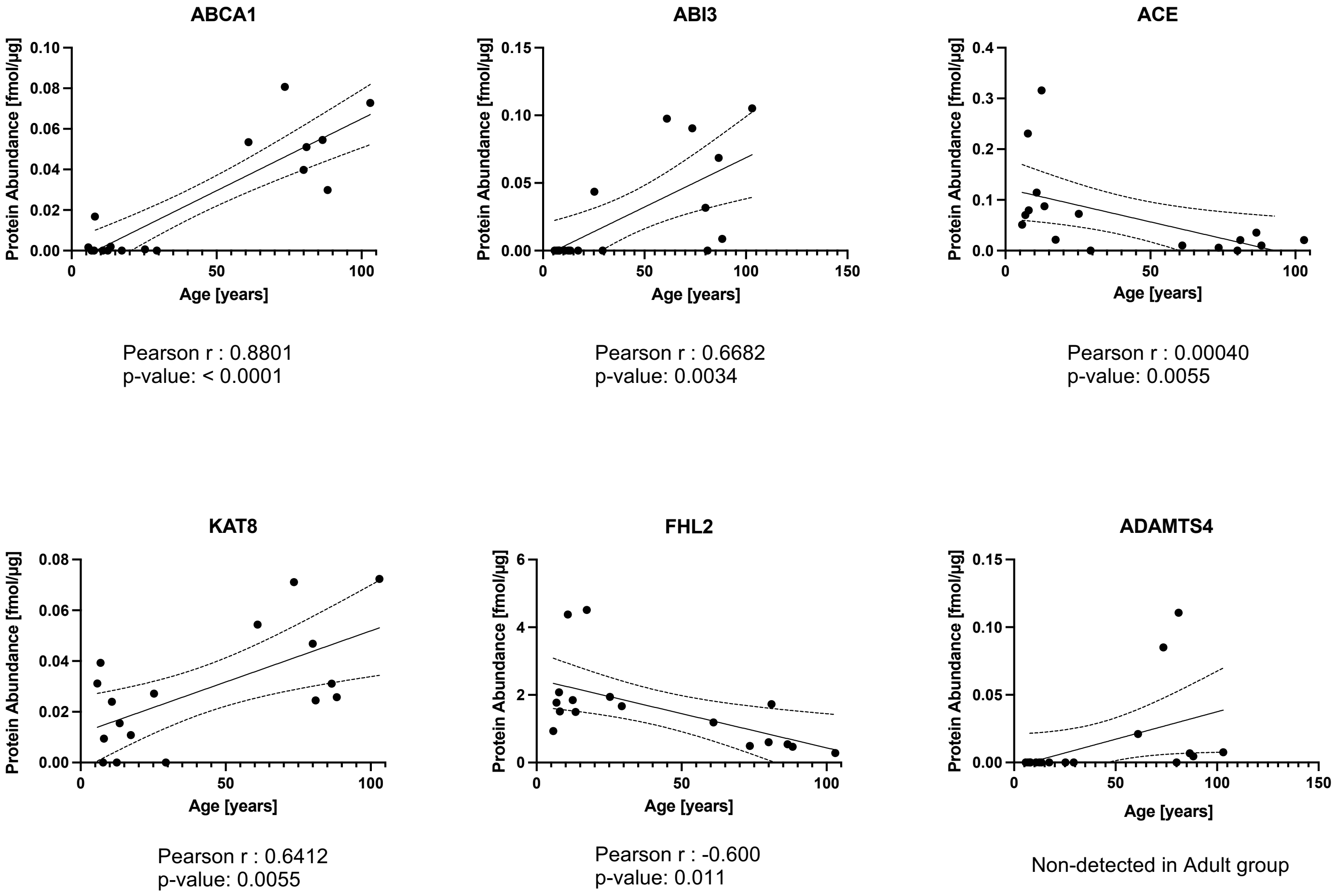

Supplemental Figure 7. Correlation plot of AD GWAS protein expression with age during the aging stage
Protein abundance of 6 AD GWAS proteins through aging are described as simple linear regression model. Individual curve are presented in (solid lines). Dashed lines represent the 95% confidence bands.

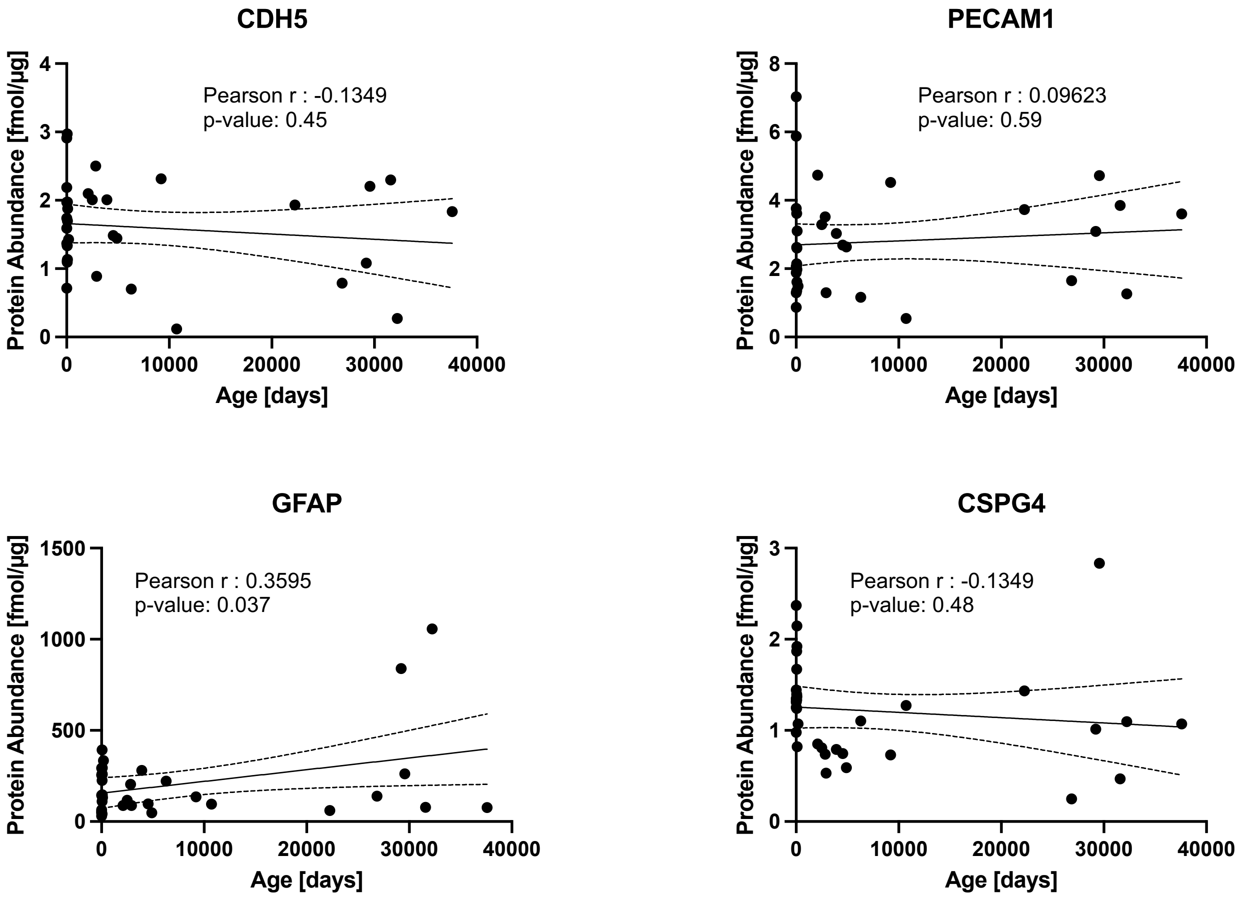

Supplemental Figure 8. Correlation plot of brain endothelial cell, astrocyte and pericyte marks with age
Protein abundance of endothelial cell marker (CDH5, PECAM1), astrocyte(GFAP) and pericyte(CSPG4) marks through aging are described as simple linear regression model. Individual curve are presented in (solid lines). Dashed lines represent the 95% confidence bands.

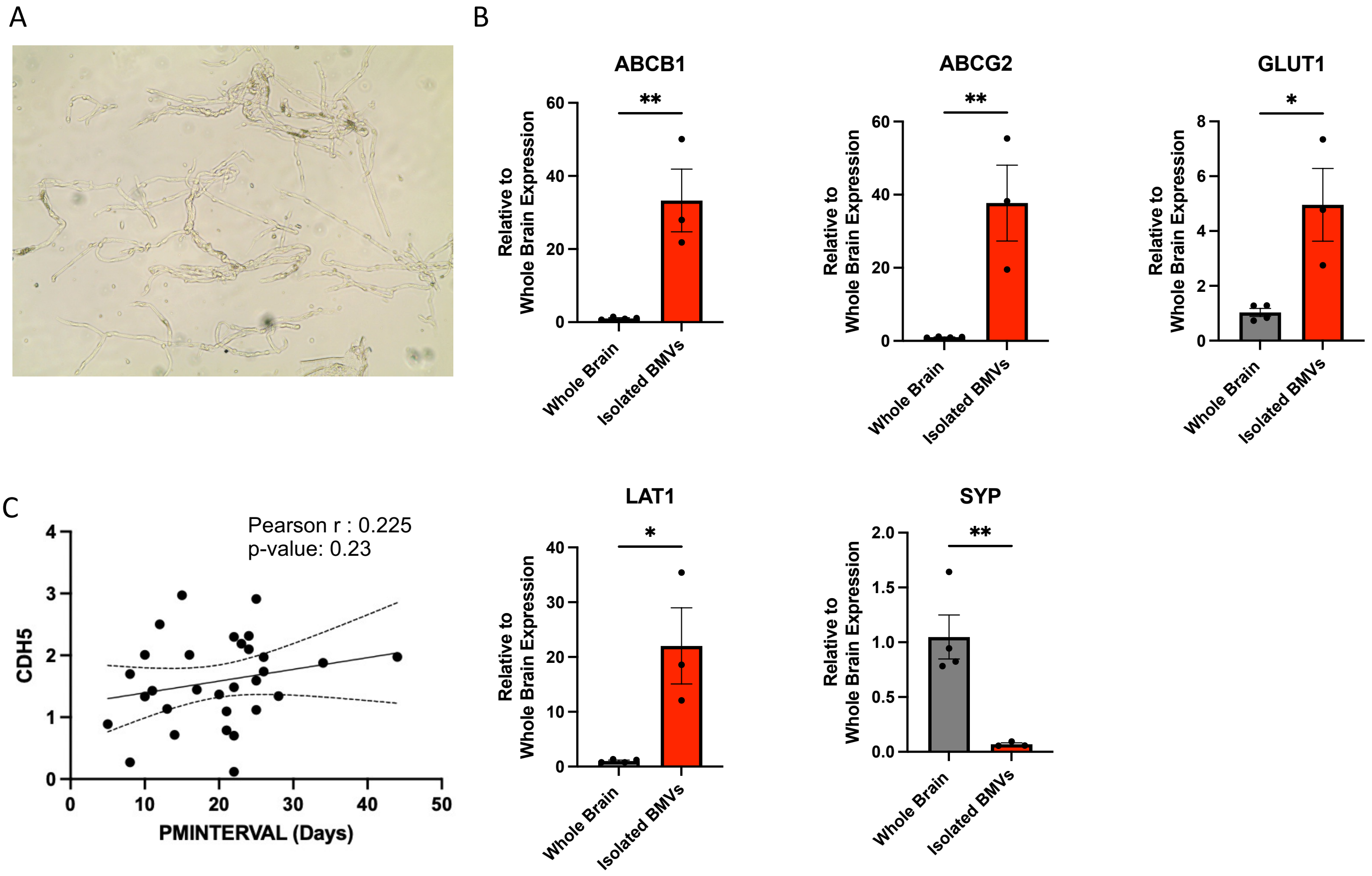

Supplemental Figure 9. Brain endothelial cell markers are enriched in isolated brain micro-vessel samples
(A)Representative microscopic images of isolated brain microvessels are shown to illustrate the morphology and purity of the isolates. (B)Fold change in mRNA expression of brain endothelial cell markers (GLUT1, LAT1, ABCB1, ABCG2) and the neuronal marker (SYP) in isolated brain microvessels (BMVs) compared to whole cortex samples. (C) Correlation between postmortem interval and representative BBB marker CDH5 is described as simple linear regression model. Individual curve are presented in (solid lines). Dashed lines represent the 95% confidence bands.

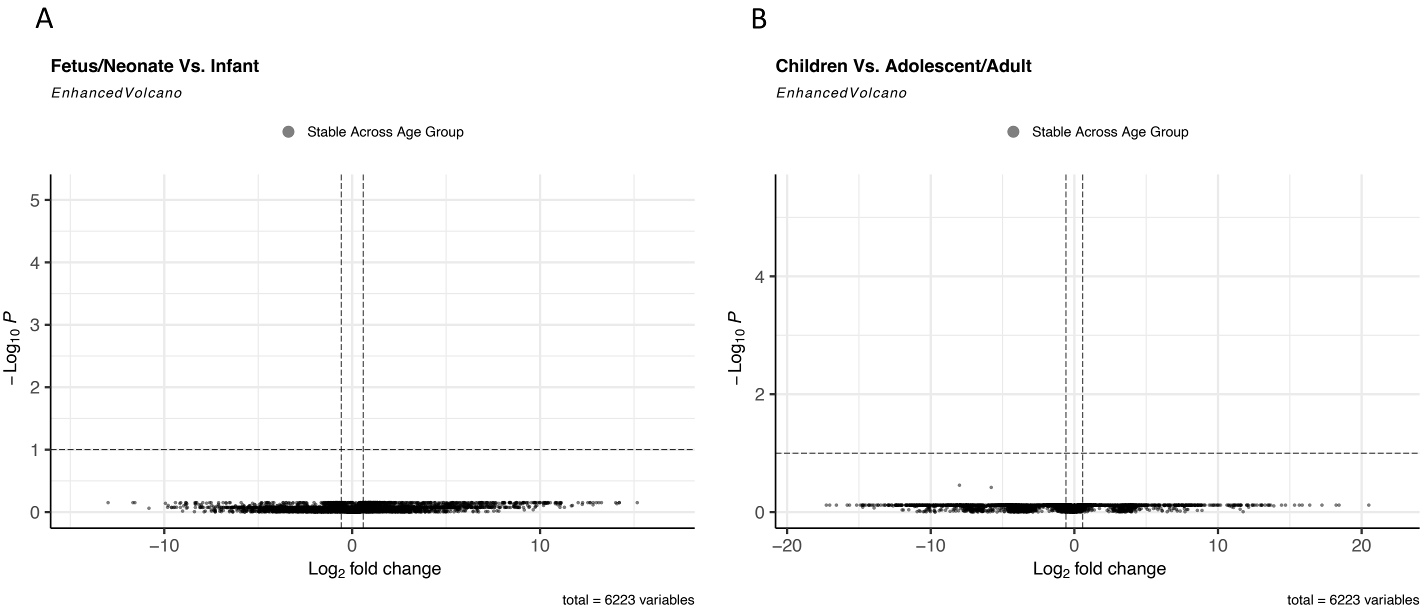

Supplemental Figure 10. Differential protein analysis showed no significantly differentially expressed proteins between the fetus/neonate and infant groups, as well as between the child and adolescent/adult groups.
(A) Volcano plot showed no proteins differentially expressed between fetus/neonate group (N=7) and infant group (N=10) of the BMV proteome. (B) Volcano plot showed no proteins differentially expressed between child group (N=5) and adolescent/adult group (N=5) of the BMV proteome. Significance is defined as absolute log2 fold change > log2(1.5) with P-value < 0.1 .
